# MazF-induced Growth Inhibition and Persister Generation in Escherichia coli*

**DOI:** 10.1074/jbc.M113.510511

**Published:** 2013-12-27

**Authors:** Arti Tripathi, Pooja C. Dewan, Shahbaz Ahmed, Raghavan Varadarajan

**Affiliations:** ‡ Molecular Biophysics Unit, Indian Institute of Science, Bangalore 560 012, India; § Jawaharlal Nehru Center for Advanced Scientific Research, Jakkur P. O., Bangalore 560 004, India

**Keywords:** Antibiotics, Cell Death, Mutant, Protein Degradation, Protein Synthesis, Toxin-Antitoxin, Inactive, Active-site Mutant, Bacteriostasis, Antibiotic Tolerance

## Abstract

Toxin-antitoxin systems are ubiquitous in nature and present on the chromosomes of both bacteria and archaea. MazEF is a type II toxin-antitoxin system present on the chromosome of *Escherichia coli* and other bacteria. Whether MazEF is involved in programmed cell death or reversible growth inhibition and bacterial persistence is a matter of debate. In the present work the role of MazF in bacterial physiology was studied by using an inactive, active-site mutant of MazF, E24A, to activate WT MazF expression from its own promoter. The ectopic expression of E24A MazF in a strain containing WT *mazEF* resulted in reversible growth arrest. Normal growth resumed on inhibiting the expression of E24A MazF. MazF-mediated growth arrest resulted in an increase in survival of bacterial cells during antibiotic stress. This was studied by activation of *mazEF* either by overexpression of an inactive, active-site mutant or pre-exposure to a sublethal dose of antibiotic. The MazF-mediated persistence phenotype was found to be independent of RecA and dependent on the presence of the ClpP and Lon proteases. This study confirms the role of MazEF in reversible growth inhibition and persistence.

## Teaser

**Background:**

MazEF is a chromosomally encoded bacterial toxin-antitoxin system whose cellular role is controversial.

**Results:**

Expression of chromosomal MazF inhibits cell killing by multiple antibiotics in a Lon and ClpP dependent manner.

**Conclusion:**

MazF is involved in reversible growth inhibition and bacterial drug tolerance.

**Significance:**

Inactive, active-site toxin mutants yield functional insights by selectively activating the corresponding WT toxin *in vivo*.

## Introduction

Free-living bacteria are exposed to fluctuating environmental conditions. Bacteria have evolved different elements/genes that regulate the rate of various intracellular metabolic processes and help bacteria survive under diverse conditions. Toxin-antitoxin (TA)^3^ loci have been proposed as general stress response elements in prokaryotes (1., 2., 3., 4.). TA loci were originally discovered on plasmids where they help in plasmid maintenance by killing or inhibiting the growth of plasmid-free cells (5, 6). Each TA locus consists of a pair of genes. Typically, the downstream gene encodes for a stable toxin and the upstream gene encodes for a labile antitoxin. Comprehensive genome analyses have shown that TA loci are ubiquitous in free-living prokaryotes but are less abundant in obligatory intracellular bacteria (7). For example, *Mycobacterium tuberculosis* has more than 80 TA loci, whereas *Mycobacterium leprae* has two TA pseudo loci (7). The biological function of TA loci when present on a plasmid is clear, but the roles of many chromosomal TA loci are unclear. There are different models that have been proposed to explain their presence on the chromosome. These include the programmed cell death model (PCD), the growth modulation model, the persistence model, and the development model (8). Despite a plethora of studies on TA systems, in most cases their functions remain to be elucidated. A common difficulty encountered during study of a specific TA system is functional redundancy with other TA systems present in the same organism. Many genomes contain multiple TA loci. Hence study of the *in vivo* role of a particular TA locus requires the selective activation of that specific locus in the background of other TA loci. Ectopic overexpression of the active, toxin component often leads to cell death, further complicating elucidation of TA system function. In the present study we use a method described previously (9) to study one of the best characterized *Escherichia coli* chromosomal TA systems, *mazEF* (5, 7). The MazEF TA system consists of a labile antitoxin MazE and a stable toxin MazF. They are encoded by the genes *mazE* and *mazF*, which are located downstream of the *relA* gene (10). The expression of the *mazEF* is negatively autoregulated by binding of either MazE or MazE-MazF complex to two upstream promoters (11). *In vivo*, during exponential growth, MazE is normally degraded by the ATP-dependent ClpAP serine protease (10). MazF is a sequence-specific endoribonuclease that preferentially cleaves mRNAs at the 3′ end of the first A base in an ACA sequence in a ribosome-independent manner (12, 13).

The biological role of *mazEF* is controversial. Some studies assert an important role for *mazEF* in PCD (10, 14, 15), whereas other studies refute this (3, 16, 17) and suggest that overexpression of RelE and MazF toxins does not confer cell killing (16). Rather, the toxins induce a bacteriostatic condition that can be fully reversed by expression of the cognate antitoxin. Concomitant with the loss of the ability to form colonies, RelE and MazF overexpression inhibited translation. However in both cases, subsequent overexpression of the cognate antitoxins was reported to simultaneously reverse the inhibition of macromolecular synthesis and the bacteriostatic condition. In another study (17), an *E. coli* mutant strain deleted for five known TA systems was examined under five different stress conditions, namely amino acid starvation, rifampin treatment, chloramphenicol treatment, acidic stress, and nutritional downshift and also during the post-stress recovery phase. In contrast to earlier reports, it was found that under all the tested conditions, the wild type and mutant strains showed similar reversible growth inhibition indicating that neither the growth inhibition nor the post stress recovery phase was dependent on any of the five TA systems. Because many bacteria contain multiple homologous TA systems with overlapping functions, knock-out of a single TA system often does not produce an observable phenotype (18., 19., 20.). In the present work we study the effect of a specific TA module, *mazEF*, in the background of several TA systems. We use a previously described methodology for regulating the expression of toxin in a tunable fashion (9). The method involves activation of the wild type (WT) toxin by overexpression of a mutated version of the toxin that has an unaltered affinity for the antitoxin but a very low affinity/activity for the cellular target of WT toxin (9). Relative to ectopic expression from a heterologous promoter, our method has the following advantages. First, WT MazF is expressed from its cognate promoter. Second, although there are multiple systems for low and tunable expression in *E. coli*, this is not true for other bacteria. Third, at low levels of induction from a tightly regulated promoter, there can be issues with cell to cell variability in expression. This is not an issue in our approach, where high inducer concentrations are used to overexpress the inactive, active-site mutant. Using this methodology we show that MazF is involved in inhibiting bacterial cell growth and enhancing drug tolerance/bacterial persistence rather than cell killing. Persisters are enriched in a dormant, slow growing fraction of cells present and are genetically identical to the remainder of an actively growing bacterial population. Persisters are tolerant to various drugs in the absence of any specific, active resistance mechanism (21., 22., 23.). In addition, whereas previous studies have demonstrated overexpression of the *mazEF* genes in persister cells (18, 24), the specific contribution of the MazEF system in generation of persisters had not been determined. In the present work, we conclusively show that MazF plays a definite role in generation of persisters that is dependent on activation by ClpP and Lon proteases and independent of the RecA mediated SOS response.

## EXPERIMENTAL PROCEDURES

##### Bacterial Strains, Plasmids, and Mutagenesis

*E. coli* host strains and plasmids used for this study are mentioned in Table 1. BW25113 (WT) is a wild type *E. coli* K12 strain that has the *mazEF* operon present on the chromosome. JW2753-1 (WT) is a derivative of BW25113 in which the *mazF* gene has been deleted from the chromosome, and this is referred to BWΔmazF. JW4184–2 is a derivative of BW25113 in which the *chpB* gene (*mazF* homolog) has been deleted from the chromosome, and this is referred to BWΔchpB. The strains BWΔclpP, BWΔlon, and BWΔrecA are BW25113 strains that have deletions of *clpP*, *lon*, and *recA* genes, respectively, from the chromosome. Strains BW25113, BWΔmazF, BWΔchpB, BWΔclpP, BWΔlon, and BWΔrecA were obtained from the *E. coli* genetic Stock Center at Yale (CGSC). Strains BWΔmazF, BWΔclpP, BWΔlon, and BWΔrecA were made markerless by using the plasmid pCP20 expressing Flippase recombinase (25). MCΔmazF and MGΔmazF strains are derivatives of *E. coli* strains MC4100 and MG1655, respectively. Both strains have a deletion of the *mazF* gene from the chromosome. MazF mutant genes were cloned between NdeI and HindIII sites in plasmid pBAD24. The *mazF* gene was amplified from the genome of BW25113 strain (WT) of *E. coli*, and the mutants were generated by a mega-primer-based method of site-directed mutagenesis as described previously (26). The identity of the cloned mutants was confirmed by DNA sequencing. The plasmid pRK9 (a derivative of pBAD33 vector) expressing the degradable GFP under control of the P_BAD_ promoter was kindly provided by Prof. John E. Cronan (University of Illinois, Urbana, IL). MC4100 strain was kindly provided by Dr. Abhijit A. Sardesai (Centre for DNA Fingerprinting and Diagnostics, Hyderabad, India). The presence of the *relA* gene in this strain was confirmed by growth of the strain on SGM minimal media containing 0.2% glucose and 40 μg/ml of the three amino acids (serine, glycine, and methionine) (27).

**TABLE 1:** *E. coli* strains and plasmids used in this study Amp^R^ and Cm^R^ indicate ampicillin and chloramphenicol resistance, respectively.

Strains and plasmids	Genotype/relevant characteristics	Source/Ref.
**Strains**		
MG1655	*mazEF, clpP, lon, recA*	A. A. Sardesai
MG1655ΔmazEF	MG1655, Δ*mazEF*::*cat*	A. A. Sardesai
MGΔmazF	MG1655, Δ*mazF*	This work
BW25113	*mazEF, clpP, lon, recA*	Baba *et al.* (30)
ΒWΔmazF	ΒW25113, Δ*mazF, clpP, lon, recA*	Baba *et al.* (30)
BWΔchpB	ΒW25113, Δ*chpB, clpP, lon, recA*	Baba *et al.* (30)
ΒWΔclpP	ΒW25113, *mazEF*, Δ*clpP, lon, recA*	Baba *et al.* (30)
BWΔlon	ΒW25113, *mazEF, clpP*, Δ*lon, recA*	Baba *et al.* (30)
ΒWΔrecA	ΒW25113, *mazEF, clpP, lon*, Δ*recA*	Baba *et al.* (30)
MC4100	*mazEF, clpP, lon, recA*_,_ relA	A. A. Sardesai
MCΔmazF	MC4100, Δ*mazEF, clpP, lon, recA*_,_ relA	This work

**Plasmids**		
pBAD24WTmazF	Amp^R^ , P_BAD_ promoter, *mazF*	This work
pBAD24E24AmazF	Amp^R^ , P_BAD_ promoter, *E24AmazF*	This work
pRK9	Cm^R^, P_BAD_ promoter, medium degradable *gfp* gene	Morgan-Kiss *et al.* (44)
pCP20	Amp^R^, Flippase recombinase	Cherepanov and Wackernagel (25)
pET15bmazE	Amp^R^, *mazE*, T7 promoter	This work
pBAD24mazE	Amp^R^, *mazE*, P_BAD_ promoter	This work

##### Construction of MCΔmazF Strain

A P1 lysate was prepared on a donor *E. coli* strain MG1655ΔmazEF containing an interruption of the *mazEF* operon by a *cat* gene from pKD3 plasmid (Δ*mazEF*::*cat*). A recipient strain, MC4100, was infected with prepared lysate and plated on LB-agar medium containing 25 μg/ml chloramphenicol as described previously (28). Positive colonies containing the *cat* cassette at the *mazEF* locus were confirmed by both PCR and DNA Sanger sequencing. The strain MCΔ*mazEF*::*cat* was made markerless by excising the *cat* gene after transforming the strain with the pCP20 plasmid expressing Flippase recombinase. The resulting strain is referred to as MCΔmazF (29).

##### Construction of MGΔmazF Strain

The *kan* cassette containing FRT site at both ends was PCR-amplified from the BWΔmazF::kan strain containing a *kan* gene from pKD4 plasmid at the place of the *mazF* gene in BW25113 (29, 30). An *E. coli* K12 strain, MG1655 transformed with pKD46 plasmid, was electroporated with amplified *kan* cassette containing ∼50 bp at each end that was homologous to the flanking sequences of the *mazF* gene. Transformants were screened on kanamycin (50 μg/ml) containing LB agar plates. The presence of the *kan* cassette at the *mazF* locus was confirmed by PCR. The resulting strain, MGΔmazF::kan, was made markerless by transforming the strain with pCP20 expressing Flippase recombinase, and the resulting strain was named MGΔmazF (25, 29).

##### Bacterial Growth Monitored as a Function of Arabinose Concentration

*E. coli* strains WT and BWΔmazF (WT with deletion of Δ*mazF* gene on chromosome) were transformed in parallel with pBAD24 plasmids expressing WT MazF, MazF (E24A), MazF (H28A), and wild type Trx under control of the arabinose inducible P_BAD_ promoter. 5 μl of the transformation mix were grown overnight in 5 ml of LB/amp (100 μg/ml). Wherever not indicated, the growth of culture has been carried out at 37 °C with shaking at 180 rpm. 50 μl of a 10^−4^ dilution was plated on six-well LB-amp plates containing either 0 or 0.2% arabinose.

##### Construction of pET15bmazE and pBAD24mazE Plasmids

To construct plasmid, pET15bmazE, pET15b-6×his-gyrA-3×FLAG plasmid was digested with NotI and NdeI restriction enzymes and linearized by removing the *gyrA* gene. The linearized vector has a T7 promoter at one end and a 3× FLAG tag sequence (5′-gattataaagatcatgat-3′) at the other end. The *mazE* gene was PCR amplified from the *E. coli* strain BW25113 containing WT *mazEF* operon using forward primer and reverse primers that have ∼30 bp homologous sequences at the each end to the linearized vector. The PCR product and the vector were recombined by the Gibson assembly method (31). After recombination, *E. coli* strain DH5α was transformed with the recombined product, and positive transformants were screened by colony PCR using vector-specific forward and gene-specific reverse primers. Further confirmation of the clones was done by DNA sequencing, and the resulting clone was named as pET15bmazE. This has 6× histidine and 3× FLAG tags at the 5′ and 3′ ends of the *mazE* gene, respectively. For the cloning of 6× histidine-*mazE-3*× FLAG into pBAD24 vector, plasmid pET15bmazF was used as a template for PCR. Gibson assembly was done to recombine NdeI- and HindIII-digested pBAD24 vector and PCR-amplified product, respectively. Positive transformants were confirmed by colony PCR and by Western blot analysis of cell lysates using peroxidase-conjugated anti-His antibody. Clones were further confirmed by DNA sequencing, and the resulting plasmid was named as pBAD24mazE.

##### Analysis of Degradation of MazE in Protease-deleted Strains

To study the effect of Lon and ClpP on the stability of MazE, WT (BW25113 strain), BWΔclpP (BW25113 lacking *clpP* gene), and BWΔlon (BW25113 lacking *lon* gene) strains were transformed with pBAD24mazE plasmid and grown in LB medium at 37 °C, 180 rpm overnight. 1% of the overnight culture was used for secondary inoculation and grown further under similar conditions to an *A*_600_ of 0.5. Cells were induced with 0.001% (w/v) arabinose for 2 h. After 2 h, inducer (arabinose) was removed, and the repressor (glucose) was added at a final concentration of 2% (w/v) to repress the further expression of MazE from the P_BAD_ promoter. Where required, heat shock was given at 48 °C for 20 min followed by incubation at 37 °C. Aliquots were taken at intervals of 30 min, and the cellular level of MazE was monitored by SDS-PAGE and quantitated using Bio-Rad ChemiDoc^TM^ MP image software, Image Lab 4.1.

##### Analysis of Transcription from mazEF Operon

*E. coli* strains MG1655 (WT strain, contains WT *mazE* and *mazF* genes on the chromosome) and MGΔmazEF (MG1655 with deletion of *mazE* and *mazF* genes from the chromosome) were transformed with pBAD24E24AmazF or pBAD24trx. Primary cultures for both the strains were grown at 37 °C for 16 h in LB media containing ampicillin (100 μg/ml) with shaking at 180 rpm. Secondary inoculation was done at a 1:100 dilution, and cells were grown at 37 °C until an *A*_600_ of 0.3 was reached followed by induction at an inducer concentration of 0.2% (w/v) for 1 h. Total RNA was purified using GE Healthcare illustra RNAspin Mini RNA Isolation kit, and cDNA from the total RNA was prepared using M-MLV reverse transcriptase (Promega). A real-time PCR analysis to quantitate expression from the *mazEF* operon was done using *mazE*-specific primers and with iQ SYBR Green Supermix at 55 °C. As an internal control, 16 S rRNA was used for normalization of expression across all the samples. To check the specificity of expression from the *mazEF* operon in the presence of an overexpressed E24A MazF mutant, mRNA levels of an unrelated gene *trx* cloned under the P_BAD_ promoter were also measured. The change in expression of *mazEF* operon from its native promoter was quantified and compared in induced and uninduced E24A MazF samples, respectively.

##### Purification and Coupling of MazE on Affi-Gel 10

*E. coli* strain BWΔmazF containing pBAD24mazE plasmid was grown at 37 °C, 180 rpm for 16 h in LB media containing ampicillin (100 μg/ml). Secondary inoculation was done with 1% of primary culture, grown further under similar conditions, and induction was done at an *A*_600_ of 0.5 with 0.2% (w/v) arabinose for 2 h. Cells were pelleted down at 5000 rpm at 4 °C, resuspended in Tris buffer (0.2 m Tris, pH 7.5, 0.5 mm EDTA, 0.5 m sucrose), and lysed by sonication at 45% amplitude for 10 min with 2 s pulse on and 8 s off. Cell lysate was centrifuged at 15,000 rpm at 4 °C to pellet down cell debris. The supernatant was incubated with nickel nitrilotriacetic acid beads for 5 h at 4 °C followed by washing (50 mm imidazole, 0.05 m Tris, pH 8.0, 0.5 m NaCl) and elution by elution buffer (500 mm imidazole, 0.05 m Tris, pH 8.0, 0.5 m NaCl). Purified protein was dialyzed in coupling buffer (0.05 m sodium bicarbonate, 0.5 m NaCl, pH 8.3) and coupled to Bio-Rad Affi-Gel 10 by using amine coupling chemistry. The amount of protein bound to Affi-Gel was estimated using fluorescence spectroscopy. Approximately 80% of the initial amount of MazE was coupled to the beads.

##### Purification of WT MazF and E24A MazF

*E. coli* strain BWΔmazF transformed with pBADE24AmazF was grown at 37 °C for 16 h in LB media containing ampicillin (100 μg/ml). Secondary inoculation was done at a dilution of 1^:^100, and cells were grown at 37 °C to an *A*_600_ of 0.5 followed by induction at an inducer concentration of 0.2% (w/v) for 1 h. Cells were pelleted down at 5000 rpm at 4 °C, and cells were lysed by sonication at 45% amplitude for 10 min with a 2-s pulse and 8 s off. Cell lysate was centrifuged at 15,000 rpm to pellet down cell debris, and supernatant was incubated with MazE conjugated beads for 5 h at 4 °C. This was followed by washing with coupling buffer and elution with glycine HCl buffer (0.2 m, pH 2.5). A similar protocol was followed for the purification of WT MazF using BWΔmazF cells transformed with pBAD24WTmazF plasmid. Protein amounts were quantified using Bio-Rad ChemiDoc^TM^ MP image software, Image Lab 4.1. Yields for E24A MazF and WT MazF were 0.4 and 0.004 mg/liter, respectively.

##### Endoribonuclease Assay

Total RNA from the *E. coli* strain MG1655 was purified using GE Healthcare illustra RNAspin Mini RNA Isolation kit. This was used as a substrate for WT and E24A MazF. RNase inhibitor was added to a final concentration of 1 unit/μl to inactivate RNase A (32). MazF (0.01 or 0.05 μg) was incubated with 6 μg of RNA in PBS buffer, pH 7.4, at 37 °C for 45 min. The reaction mixture was subsequently analyzed by agarose gel electrophoresis, and the extent of RNA degradation was quantitated using Bio-Rad ChemiDoc^TM^ MP image software, Image Lab 4.1.

##### Kinetics of Bacterial Growth Revival Monitored on Glucose-containing Medium

WT and BWΔmazF transformed with pBAD24E24AmazF were grown overnight in LB/amp. A 1:100 dilution of the saturated overnight culture was grown to an *A*_600 nm_ of 0.2. An aliquot of the culture was withdrawn, and 10-fold serial dilutions were plated on 0.2% (w/v) glucose plates. To each of the remaining cultures, arabinose was added to a final concentration of 0.2% (w/v). Subsequently, 500-μl aliquots were withdrawn at different time points and centrifuged at 4000 rpm for 5 min at room temperature. Supernatant was removed, and cells were washed twice with 500 μl of LB. 10-Fold serial dilutions were prepared and plated on 0.2% glucose plates. The % survival was calculated as the ratio ((number of cfu/ml for toxin containing strain)/(number of cfu/ml for the strain lacking the toxin)] × 100.

##### Kinetics of Cell Growth Inhibition by MazF Studied by FACS

WT and BWΔmazF transformed with pBAD24E24AmazF as well as the plasmid encoding the degradable GFP (pRK9) were grown overnight in LB + amp (100 μg/ml) + chloramphenicol (34 μg/ml). After a 1:100 dilution in the same medium, cultures were grown to an *A*_600_ of 0.2 and induced with 0.2% (w/v) arabinose. Subsequently, aliquots were withdrawn at different time points, and flow cytometry was performed on a BD FACs Scan analyzer equipped with an argon laser (emission at 488 nm/15 milliwatts) and a 530–575-nm band pass filter. For each sample 30000 events were collected at a rate between 500 and 800 events per second.

##### Role of MazF in the Generation of Persisters

WT (BW25113 and MG1655) and Δ*mazF* strains (BWΔmazF and MGΔmazF) transformed with pBAD24E24AmazF were grown overnight in LB + amp (100 μg/ml). After a 1:100 dilution, cells were grown to an *A*_600_ of 0.2 and induced with 0.2% arabinose for 1 h. An aliquot was withdrawn after 1 h, washed, appropriately diluted, and plated on 0.2% glucose plates to determine the starting cfu. Each of the remaining cultures was treated with different drugs at 10× minimum inhibitory concentration (ciprofloxacin 0.4 μg/ml, cefotaxime 100 μg/ml, mitomycin C 10 μg/ml, tobramycin 25 μg/ml, and streptomycin 25 μg/ml) for 4 h. The cultures were washed twice, appropriately diluted, and plated on 0.2% glucose plates to determine the viable counts after antibiotic treatment. % survival after drug treatment was calculated for each of the strains as the ratio (cfu obtained after drug treatment/cfu obtained before drug treatment).

##### GFP Expression in MazF-mediated Persisters

To study the kinetics of GFP expression by FACS, WT and BWΔmazF transformed with both plasmid expressing the E24A mutant of MazF and pRK9 expressing the degradable GFP were grown as described above and induced with 0.2% arabinose for 1 h. After this, ciprofloxacin or cefotaxime were added to final concentrations of 0.4 and 100 μg/ml, respectively. Aliquots were withdrawn at 1-h intervals and analyzed on a Beckman-Coulter flow cytometer (Beckman Instruments) as described above.

##### Activation of MazF by Sublethal Antibiotic or Heat Prestress

Overnight cultures of all *E. coli* strains, BW25113 (containing WT *mazF* gene), BWΔmazF (BW25113, lacks *mazF* gene), MC4100 (contains WT *mazF* gene), MCΔmazF (MC4100, lacks *mazF* gene), MG1655 (contains WT *mazF* gene), and MGΔmazF (MG1655, lacks *mazF* gene), were diluted 100-fold and grown until an *A*_600_ of 0.2–0.3. Subsequently, cells were exposed to two types of sublethal prestress, either exposure of cells to heat (48 °C for 20 min) or a sublethal dose of antibiotics (ampicillin (2.5 μg/ml for BW25113, BWΔmazF, MC4100, and MCΔmazF and 5 μg/ml for MG1655 and MGΔmazF) or ciprofloxacin (0.008 μg/ml)). This was followed by exposure of cells to various antibiotics at lethal doses (cefotaxime 100 μg/ml, ciprofloxacin 0.4 μg/ml, mitomycin C 10 μg/ml, tobramycin 25 μg/ml, and streptomycin 25 μg/ml) for 4 h. Culture was washed twice with LB and plated on LB agar media and grown for 14–16 h. The next day, colonies were counted on a plate, and % survival for each of the strains was calculated. % survival is defined as the ratio (cfu after antibiotic exposure/cfu before antibiotic exposure) ×100. The survival ratio was defined as (% survival of *mazF* containing strain/% survival of corresponding Δ*mazF* reference strain).

##### Isolation of Persisters in Strains Deleted for clpP, lon, and recA

The strains BWΔclpP (BW25113 strain containing deletion of *clpP* gene), BWΔlon (BW25113 strain containing deletion of *lon* gene), and BWΔrecA (BW25113 strain containing deletion of *recA* gene) transformed with pBAD24E24AmazF plasmid were grown overnight in LB + amp (100 μg/ml). Following 1:100 dilution, cells were grown to an *A*_600_ of 0.2 and induced with 0.2% (w/v) arabinose for 1 h. Cells were exposed to antibiotics as described in the previous section and plated on 0.2% (w/v) glucose containing LB-agar media. % survival was calculated as described above. Survival ratio is defined as the ratio of % survival of the *E. coli* strain expressing E24A MazF to the corresponding uninduced strain.

##### Minimum Inhibitory Concentration Determination

All strains were grown overnight in LB medium and diluted to a density of 5 × 10^5^ cfu/ml in fresh LB medium containing several dilutions of an antibiotic in a deep well microtiter plate with 500 μl per well. The plate was incubated for 18 h at 37 °C with 180 rpm shaking, and the minimum inhibitory concentration was determined as described previously (33).

## RESULTS

##### Expression of Low Levels of MazF Cause Growth Arrest but Not Extensive Cell Death

MazEF is one of the best studied toxin-antitoxin systems present on bacterial chromosomes. MazF is an endoribonuclease that preferentially cleaves single-stranded RNA at ACA sequences (34). The crystal structure of an *E. coli* MazE-MazF complex has been determined (35). Residues Glu-24 and His-28 were suggested to be important for activity (36), but neither residue is involved in binding to MazE (supplemental Fig. S1). We, therefore, constructed the E24A and H28A mutants of MazF as these are likely to lack activity but retain the ability to bind the antitoxin MazE. Hence, it would be expected that both mutants should be non-toxic in a Δ*mazF* strain but should cause growth arrest/cell death when expressed in a WT strain containing a chromosomal copy of WT MazF. Both the above mutants were constructed in the plasmid pBAD24 under control of the arabinose-inducible P_BAD_ promoter.

Three different bacterial strains were initially used. These were BW25113, which has a chromosomal copy of the *mazF* and *mazE* genes (referred to as WT), JW2753-1, which is a *mazF* gene knock-out mutant of the strain BW25113 (referred to as BWΔmazF), and JW4184–2, which is a *chpB* (MazF homolog) gene knock-out mutant of the strain BW25113 (referred to as BWΔchpB). Cells transformed with pBAD24-expressing WT MazF or the mutants (E24A and H28A) were then plated in the absence and presence of 0.2% arabinose. No colonies were observed upon overexpression of either WT or H28A mutants in WT and BWΔmazF strains (supplemental Fig. S2). This confirms that overexpression of WT MazF causes cell death and suggests that H28A is an active mutant of MazF. Expression of the E24A mutant resulted in pin-sized colonies in WT *E. coli* and normal sized colonies in the BWΔmazF strain (supplemental Fig. S2). This confirms that E24A is an inactive mutant of MazF and that it retains the ability to bind MazE, thus causing growth inhibition only when a WT copy of MazF is present. To further confirm that E24A MazF is an inactive mutant, an *in vitro* study of endoribonuclease activity was performed using total RNA prepared from *E. coli* strain MG1655 (WT) as a substrate for the WT MazF and E24A MazF proteins. Both proteins were affinity-purified using MazE coupled beads, demonstrating that both proteins retain the ability to bind MazE. Substrate RNA was incubated with E24A MazF or WT MazF at 37 °C for 45 min. In the case of WT MazF, degradation of RNA was observed, whereas for E24A MazF, the RNA amount remained the same as a control where MazF protein was not added (supplemental Fig. S3). This confirms that the mutant E24A MazF is an inactive, active-site mutant that lacks the ability to cleave RNA, in contrast to WT MazF. BWΔmazF strain has a chromosomal copy of *chpB*, a homolog of MazF (39% identity). Thus, lack of any phenotypic effect after E24A overexpression in BWΔmazF strain indicates that the interaction between overexpressed E24A and MazE is specific and that the E24A mutant of MazF did not bind the antitoxin of ChpB. As a control, strains transformed with a plasmid expressing the non-toxic thioredoxin gene under control of the arabinose promoter (37) showed normal growth in both the presence and absence of arabinose. Both WT and H28A MazF-expressing plasmids when transformed in BWΔchpB resulted in cell death (data not shown) in the presence of arabinose. However, E24A MazF, when transformed in BWΔchpB, again resulted in the appearance of pin-sized colonies in the presence of arabinose. This shows that the overexpressed mutant effect seen is highly specific for MazF as it is not influenced by the absence of ChpB (MazF homolog). Because H28A appeared to have *in vivo* activity similar to WT MazF, further studies were restricted to the E24A mutant of MazF.

**FIGURE 1:**
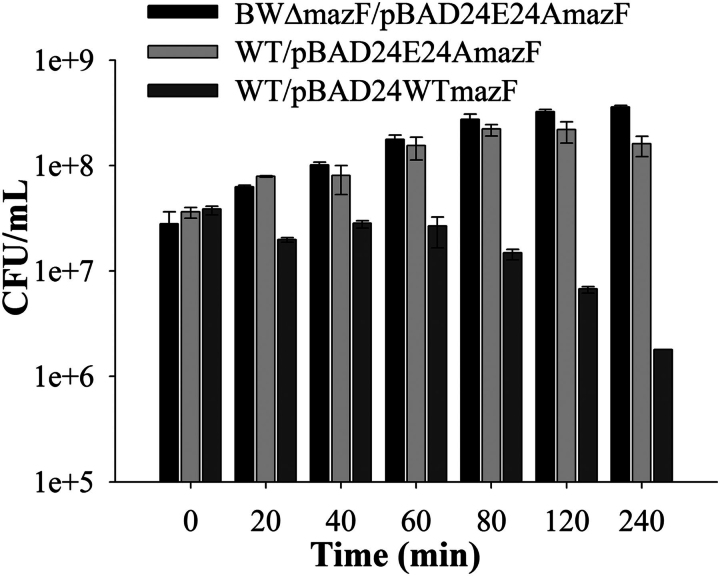
**Effect of toxin expression on cell viability as a function of time.** Overnight cultures of WT and BWΔmazF strains expressing plasmid-borne E24A MazF or WT MazF were diluted 100-fold and grown in LB medium. Cells were induced at an *A*_600_ of 0.2 with 0.2% arabinose and incubated for varying times. An aliquot was withdrawn, washed, and plated on 0.2% glucose-containing plates to repress E24A MazF or WT MazF expression from plasmid. Overexpression of E24A MazF shows reversible growth inhibition in WT *E. coli* strain but had no effect in the BWΔmazF strain. Overexpression of WT MazF results in significant cell death in both strains. Data for BWΔmazF*/*pBAD24WTmazF are not shown. The *y* axis is shown in log scale.

As mentioned above, the involvement of MazF in cell death is controversial. To resolve whether MazF is involved in cell killing or in reversible growth inhibition, the ability of cells to recover after transient expression of WT MazF was examined. Log phase cultures of WT and BWΔmazF transformed with pBAD24E24AmazF plasmid were transiently induced by the addition of 0.2% arabinose for varying amounts of time and then plated on plates containing 0.2% glucose to repress expression of the E24A mutant. In this situation as described previously (9), overexpressed E24A will titrate out cellular MazE, both releasing WT MazF and derepressing the *mazEF* operon, resulting in fresh synthesis of both MazE and MazF. However, the freshly synthesized MazE would still be titrated out by the excess E24A MazF. Upon the addition of glucose, E24A expression will cease and eventually there will be sufficient WT MazE to titrate WT MazF. The data are summarized in Fig. 1 and show that cells remain viable even after several hours of E24A MazF overexpression. However, after transient E24A MazF overexpression, colonies took 14–24 h to appear instead of the usual 8 h. The data clearly indicate that MazF is involved in reversible inhibition of cell growth and possibly transforming cells to a dormant state. Previously, MazF-mediated PCD was observed in an *E. coli* strain MC4100 (10, 14), and activation of *mazEF* was observed under various stress conditions including antibiotics (38). Hence, the activation of *mazEF* by use of an inactive, active-site mutant E24A MazF was studied by transforming WT (MC4100, contains *mazF* gene) and MCΔmazF (MC4100 derivative that lacks *mazF* gene) strains with plasmid encoding E24A MazF. Similar to the result observed in strain BW25113, MazF expression induced bacteriostasis rather than cell death in MC4100 also, whereas no bacteriostasis was seen in MCΔmazF. These results show that transcriptional activation of *mazEF* induces cells to a dormant state that can be reversed by stopping expression of the inactive, active-site mutant. Previous studies (15, 38., 39., 40.) suggest that *mazEF*-mediated PCD depends on the activation of *mazEF* through antibiotics. To study this, both WT and MCΔmazF strains were grown in the presence of ampicillin (100 μg/ml), rifampicin (15 μg/ml in M9 and 25 μg/ml in LB), or spectinomycin (200 μg/ml) for 10 and 60 min in LB and M9 media, respectively. Both WT and MCΔmazF strains showed similar patterns of growth under identical conditions (supplemental Fig. S4). Thus deletion of *mazF* does not result in decreased cell death in the presence of antibiotics. This is consistent with data from one of the studies (17) but not with other data (15, 38., 39., 40.). Overall the data strongly indicate that MazF activation leads to reversible growth inhibition rather than antibiotic-mediated PCD.

##### Activation of mazEF Operon by an Inactive, Active-site Mutant

**FIGURE 5:**
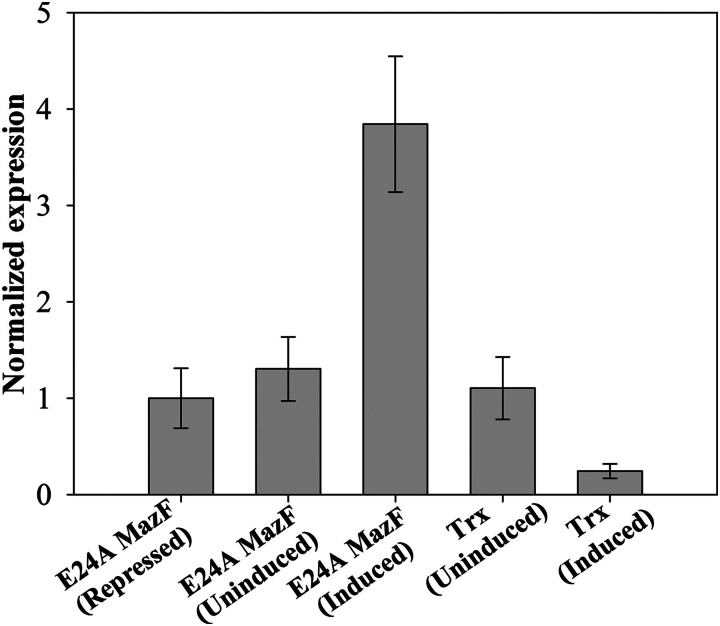
**Activation of *mazEF* operon upon induction of an inactive, active-site mutant E24A MazF.** Both *E. coli* strains, MG1655 (contains WT *mazEF* operon on chromosome) and MGΔmazEF (lacks *mazEF* operon on chromosome), were induced for the expression of E24A MazF from the pBAD24 plasmid with 0.2% (w/v) arabinose for 1 h, total RNA was prepared and converted to cDNA, and quantitative PCR was performed to detect the mRNA specific to *mazE*. Expression of the *mazEF* operon was compared in samples from identical cells grown in either the absence of inducer (arabinose) or presence of repressor (glucose). The *y* axis represents normalized expression of the *mazEF* operon under different conditions. The *x* axis represents the different conditions under which MG1655 cells were grown. As a control for the specificity of E24A MazF for *mazEF* operon activation, transcription of *mazEF* operon in the presence of an unrelated protein, Trx was monitored. The overexpression of E24A MazF causes the derepression of the *mazEF* operon in WT MG1655 strain, and even uninduced cells have slightly higher levels of *mazEF* operon activation compared with cells repressed for E24A MazF. Lack of activation of *mazEF* in uninduced and induced Trx cells shows that the effect of E24A MazF is specific to the *mazEF* operon.

E24A MazF is an inactive, active-site mutant that can bind to cognate antitoxin but lacks toxic activity. However, when this mutant is expressed in cells containing a WT *mazEF* operon, it elicits a toxic response, probably by the release of WT MazF from the MazE-MazF complex and/or activating the operon for the fresh synthesis of MazE and MazF, respectively. Mutant E24A MazF then binds to freshly synthesized MazE, and WT MazF is free to show its toxic effects. In our system overexpression of E24A MazF disturbs the *in vivo* ratio of MazF to MazE, which is very crucial in conditional transcriptional regulation for the *mazEF* operon (11), similar to other TA systems including *ccd* and *relBE* (41, 42). To study the effect of an overexpressed E24A MazF on transcription from the *mazEF* operon, quantitative PCR was performed. Total RNA was prepared from the induced and uninduced E24A MazF WT and ΔmazF cells, respectively, and converted to cDNA. and the level of expression was quantified using quantitative PCR (see Fig. 5). Relative to uninduced and repressed cells, an increased expression of the *mazEF* operon was seen in WT cells, which were induced for E24A MazF. Uninduced cells have a higher level of *mazEF* operon expression relative to cells repressed for E24A MazF expression. This could be due to leaky expression of E24A MazF from the P_BAD_ promoter. About 1000-fold lower expression of *mazEF* was found in the *mazF*-deleted strain relative to the uninduced WT, and this could be due to nonspecific amplification in this strain. To study the specificity of E24A MazF toward activation of the *mazEF* operon, an unrelated protein, Trx, was overexpressed from the same P_BAD_ promoter as a control. The expression level of the *mazEF* operon in uninduced WT cells containing pBAD24trx is the same as in repressed pBAD24 E24A *mazF* cells, indicating that there is no interference of Trx in *mazEF* operon expression. However, cells with overexpressed Trx show a low level of *mazEF* mRNA that could be due to engagement of transcriptional machinery in the overexpression of Trx from the pBAD24 plasmid. Enhancement in the expression of *mazEF* in WT cells induced for E24A MazF indicates that overexpression of E24A MazF leads to derepression of the *mazEF* operon. Hence the observed phenotypes result both from titration of MazE by an inactive, active mutant as well as derepression of transcription from the *mazEF* operon and hence fresh synthesis of WT MazF. A similar effect was observed for the *ccd* operon upon overexpression of an inactive, active-site mutant of the CcdB toxin (9).

##### Expression of Low Levels of MazF Partially Inhibits Protein Synthesis

**FIGURE 2:**
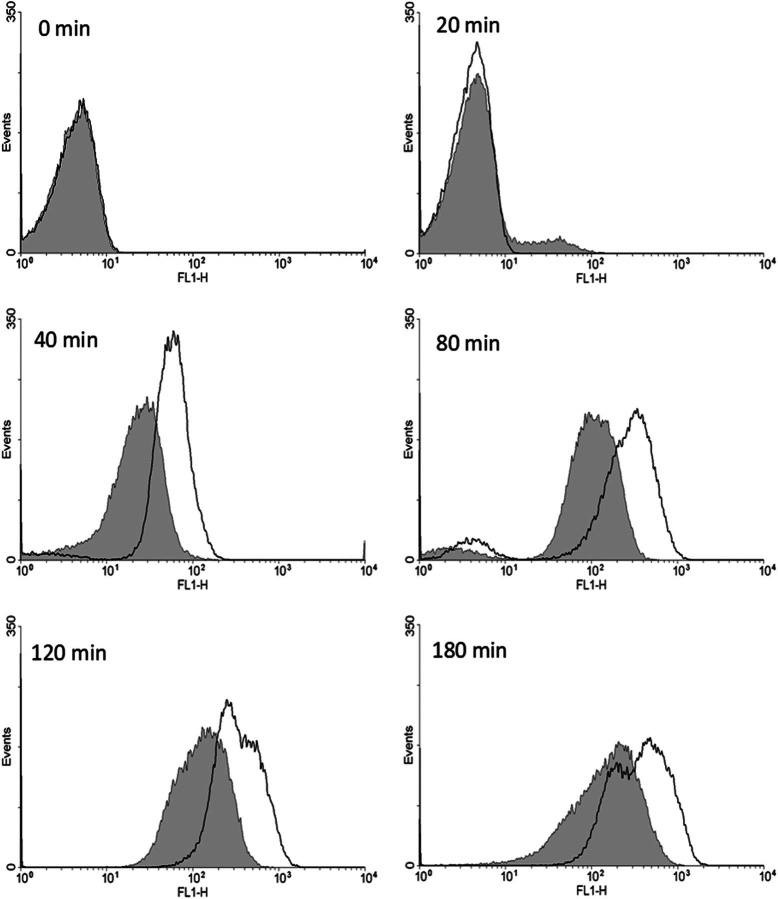
**Kinetics of protein expression in WT and Δ*mazF* strains.** Protein expression was monitored by GFP production using flow cytometry. WT (*filled curves*) and BWΔmazF (*empty curves*) strains carrying both the plasmid-borne degradable GFP as well as the E24A MazF mutant were induced at an *A*_600 nm_ of 0.2 with 0.2% arabinose. GFP expression as a function of time after induction was monitored at regular intervals using flow cytometry. Substantial GFP expression is seen in both strains even after several hours of E24A MazF expression, although there was lower expression in the WT strain.

**FIGURE 3:**
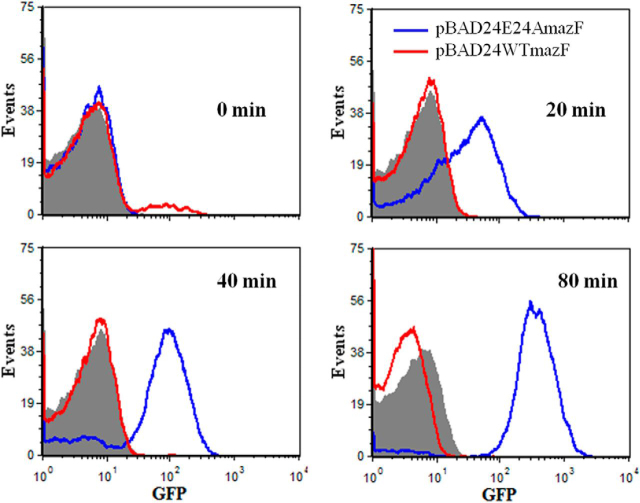
**Comparison of GFP expression in the WT strain overexpressing WT MazF from the P_BAD_ promoter and when expressing MazF from its own promoter after activation by E24A MazF.** GFP expression was monitored as a function of time by flow cytometry. WT strain transformed with pBAD24 containing either the wild type *mazF* gene or the *E24AmazF* gene was induced at an *A*_600_ of 0.2 with 0.2% arabinose. The *red* and *blue histograms* correspond to GFP expression in cells overexpressing WT MazF and E24A MazF, respectively. The *filled curve* represents the GFP uninduced control. Although overexpression of WT MazF from the P_BAD_ promoter completely abolishes GFP expression, expression of lower levels of free MazF from its cognate promoter only partially inhibited GFP expression.

It has been shown previously that toxins from TA modules such as RelE and MazF affect macromolecular synthesis (14, 16, 43). However, these studies typically involve substantial overexpression of the toxin from a heterologous promoter. To study the effects of lower levels of WT MazF expression, both WT and BWΔmazF strains containing the pBAD24E24AmazF plasmid were transformed with another compatible plasmid carrying a gene for degradable GFP (44) under control of the P_BAD_ promoter. This GFP has a *t*_½_ of ∼34 min *in vivo* (44). The data (summarized in Figs. 2 and 3) indicate that a low level of WT MazF driven by its own promoter but triggered by E24A MazF expression in the WT strain partially inhibits protein expression. However, there is still appreciable GFP expression and little cell death even after 4 h. In contrast, overexpression of the WT MazF protein from a heterologous P_BAD_ promoter resulted in almost complete shutdown of the translation machinery as monitored by GFP expression, even in the initial time points (Fig. 3).

##### MazF Protein Plays a Role in Generation of Persisters

**FIGURE 4:**
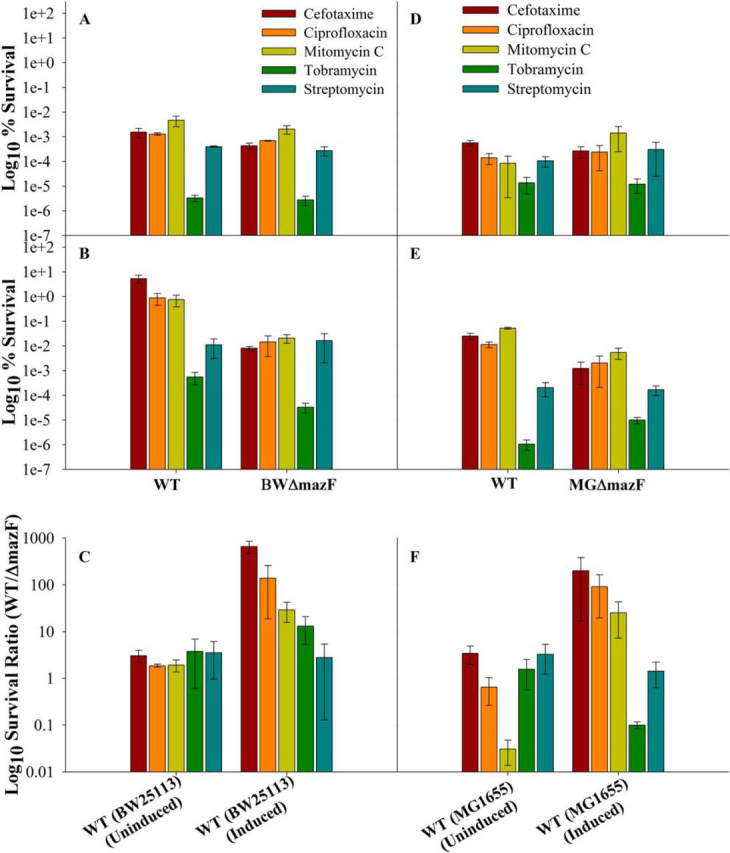
**MazF protects cells against cefotaxime, ciprofloxacin, mitomycin C, and tobramycin to decreasing extents.** WT (BW25113 and MG1655) and Δ*mazF*, (BWΔmazF and MGΔmazF) strains were induced for the expression of E24A MazF mutant protein for 1 h at an absorbance of 0.2. Subsequently the cells were challenged with different antibiotics at ∼10 times minimum inhibitory concentration for 4 h. % survival was calculated for each strain as the ratio (cfu before antibiotic exposure/cfu after antibiotic exposure) × 100. Survival ratio was defined as (% survival in WT/% survival of corresponding strain deleted for *mazF* gene). *Panels A* and *B* show the % survival for the uninduced and induced for BW25113 WT and BWΔmazF strains, respectively, and *panels D* and *E* show the % survival for the uninduced and induced for MG1655 WT and MGΔmazF strains, respectively. *Panels C* and *F* show the survival ratio of uninduced and induced for the WT strains, BW25113 and MG1655, respectively. Induced cells show higher survival compared with uninduced cells, indicating that activation of *mazF* expression from its cognate promoter can confer protection toward various antibiotics. Both % survival and survival ratio are shown in log scale. *Error bars* indicate the S.E. from three independent experiments.

The involvement of MazF in inhibition of cell growth and protein synthesis led us to examine whether release of MazF from the MazE-MazF complex *in vivo* would also result in the generation of persisters. WT and BWΔmazF strains induced for E24A expression for 1 h were subsequently exposed to lethal doses of different drugs (ciprofloxacin, cefotaxime, mitomycin C, tobramycin, streptomycin) for 4 h. Cells were then washed and plated on 0.2% glucose-containing medium to repress expression of E24A, and the surviving colonies were counted. In comparison to the BWΔmazF strain, WT strain showed ∼10, 30, 120, and 700-fold higher tolerance to tobramycin, mitomycin C, ciprofloxacin, and cefotaxime, respectively (Fig. 4). Mitomycin C and ciprofloxacin both target DNA replication, cefotaxime targets cell wall biosynthesis, and tobramycin targets protein synthesis. No protection was observed against the protein synthesis inhibitor streptomycin (Fig. 4, *panel C*).

As a control we monitored the % survival of WT cells uninduced for E24A when treated with different antibiotics and found ∼2–4-fold more survival compared with the BWΔmazF strain uninduced for E24A (Fig. 4, *panel A*). Our study thus conclusively shows that release of MazF from the MazEF complex can lead to the generation of persisters. This is consistent with earlier studies (24) which have demonstrated that controlled expression of WT MazF at low levels (0.015% arabinose) driven by the P_BAD_ promoter as well as similar overexpression of three other proteins (HipA, PmrC, DnaJ) gave rise to increased persister formation (24). Even in the absence of E24A induction, WT consistently shows increased survival relative to the BWΔmazF strain. This shows that even basal level expression of MazF is able to give protection against antibiotics; however, protection is considerably enhanced upon artificial activation of the *mazEF* TA system by overexpression of the inactive, active-site mutant, E24A MazF. In agreement with one earlier study (17) but in contrast to another (38), these data confirm that exposure to antibiotics alone does not trigger *mazEF*-mediated lethality. Instead, if sufficiently activated by other mechanisms, the *mazEF* system may protect bacteria against some antibiotics.

Because BW25113 contains multiple gene deletions (30, 45), a similar experiment with an overexpressed E24A was performed with the widely used *E. coli* K12 strain MG1655 and its derivative, MGΔmazF, which lacks the *mazF* gene. Similar to BW25113 strain, protection against cefotaxime (200-fold), ciprofloxacin (100-fold), and mitomycin C (20-fold) was observed in MG1655 WT strain induced for E24A MazF relative to the MGΔmazF strain (Fig. 4*F*). Higher survival was observed even in uninduced cells compared with cells that lacked the plasmid expressing E24A MazF (Fig. 4*F*). This again confirms that even a basal level of *mazF* expression from its native promoter, as seen in quantitative real-time reverse transcription PCR (qRT-PCR) experiments (Fig. 5) in the presence of the pBAD24E24AmazF plasmid, provides protection against various antibiotics. The effects of MazF in promoting a persister phenotype are thus observable in multiple *E. coli* strains.

##### Flow Cytometry-based Characterization of the Generated Persisters

**FIGURE 6:**
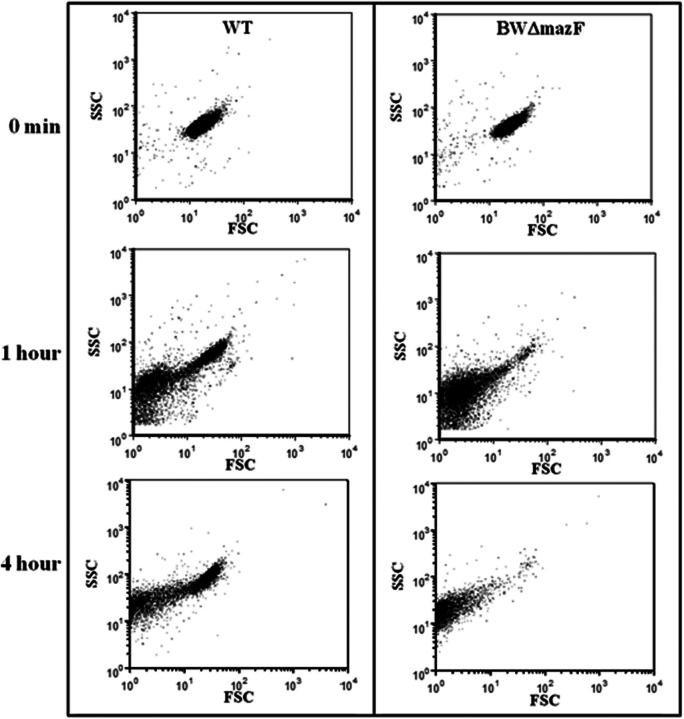
**MazF activation protects cells from lysis by cefotaxime.** FSC-SSC FACS data of WT (*left*) and BWΔmazF (*right*) strains expressing E24A MazF after different times of cefotaxime treatment are shown. Both WT and BWΔmazF strains transformed with plasmid pBAD24E24AmazF were grown to an *A*_600_ of 0.2, induced for 1 h with 0.2% arabinose, and subsequently treated with cefotaxime for 1–4 h. The change in cell size (FSC) and granularity (SSC) was monitored by flow cytometry at different time points. Almost all BWΔmazF cells were lysed after 1 h of cefotaxime treatment. In contrast, a significant fraction of WT cells remain unlysed even after 4 h of exposure to cefotaxime.

In addition to the cfu-based data showing the presence of WT cells tolerant to cefotaxime, flow cytometry also showed a clear difference among the two strains (WT and BWΔmazF) after induction of E24A MazF followed by cefotaxime treatment (Fig. 6). Whereas intact cells could be seen in the FSC-SSC plot of WT cells treated with cefotaxime after 4 h, only cell debris were observed for the BWΔmazF cells. The intact cells present after 4 h of cefotaxime treatment in WT cells showed a complete loss of GFP expression.

The kinetics of GFP production by WT and BWΔmazF cells after induction of E24A followed by ciprofloxacin treatment was also studied by flow cytometry (supplemental Fig. S5). As expected, cells without ciprofloxacin treatment showed decreased, although still significant GFP production in WT cells relative to BWΔmazF cells because of inhibition of protein synthesis by MazF. In addition, there was a time-dependent increase in the population of cells lacking GFP fluorescence in the WT strain after ciprofloxacin treatment in comparison to the untreated cells. Although the exact mechanism of persistence is not well understood, it is at present hypothesized that persisters are a metabolically dormant subpopulation of cells (21). The FACS data clearly indicated that after both ciprofloxacin and cefotaxime treatment, a fraction of cells definitely goes into a dormant state which might be correlated with the observed tolerance to the antibiotic.

##### Activation of mazEF Loci by Sublethal Doses of Different Antibiotics

**FIGURE 7:**
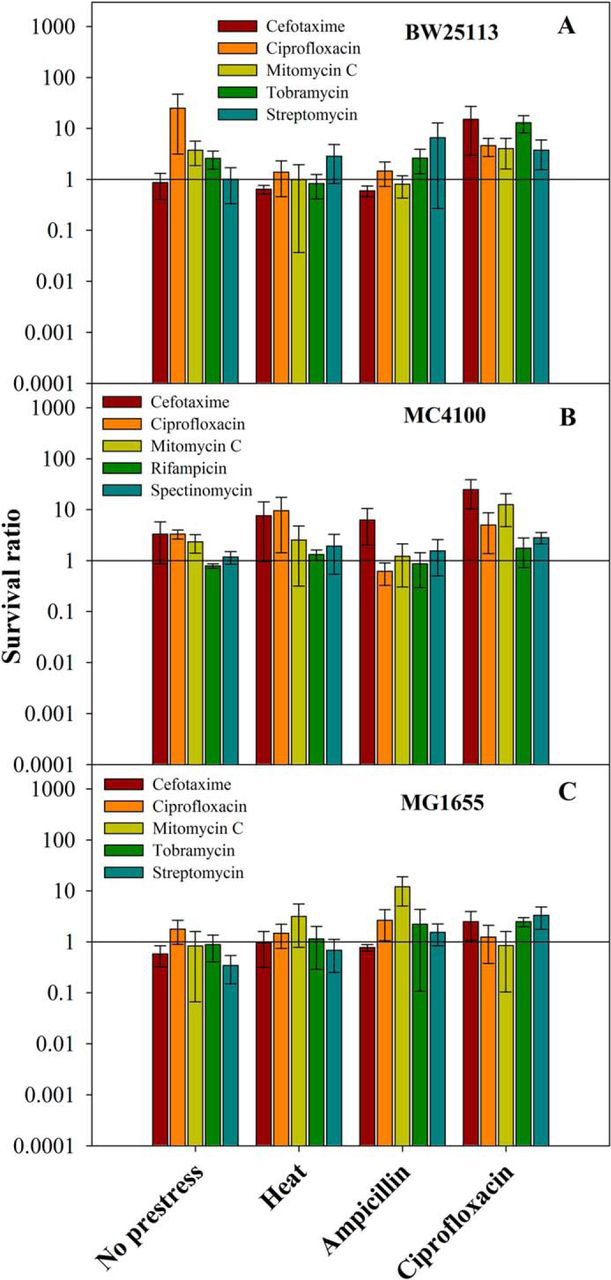
**Role of MazF in persister generation when present in three different *E. coli* strains, BW25113, MC4100, and MG1655.** Overnight culture of all *E. coli* strains BW25113 (contains WT *mazF* gene), BWΔmazF (BW25113 strain, lacks *mazF* gene), MC4100 (contains WT *mazF* gene), MCΔmazF (MC4100 strain, lacks *mazF* gene), MG1655 ((contains WT *mazF* gene), and MGΔmazF (MG1655 strain, lacks *mazF* gene) were diluted 100-fold and grown until an *A*_600_ of 0.2–0.3. Subsequently, cells were exposed to two types of sublethal prestresses, either exposure of cells to heat (48 °C for 20 min) or a sublethal dose of antibiotics (ampicillin (2.5 μg/ml for BW25113 and MC4100 and 5.0 μg/ml for MG1655)) or ciprofloxacin (0.008 μg/ml)). This was followed by exposure of cells to various antibiotics at lethal doses (cefotaxime 100 μg/ml, ciprofloxacin 0.4 μg/ml, mitomycin C 10 μg/ml, tobramycin 25 μg/ml, streptomycin 25 μg/ml, rifampicin 20 μg/ml, and spectinomycin 200 μg/ml) for 4 h. The culture was washed twice with LB and plated on LB agar media for cfu/ml determination. % survival was defined as the ratio (cfu after antibiotic exposure/cfu before antibiotic exposure) ×100. Survival ratio is defined as the ratio (% survival of the *E. coli* strain containing the *mazF* gene/% survival of corresponding strain deleted for *mazF* gene). *Panels A–C* show the survival ratio for BW25113, MC4100, and MG1655 strains, respectively. A sublethal dose of ciprofloxacin results in a small but significant increase in persister formation in all the three *E. coli* strains, whereas heat prestress enhance persisters only in MC4100 and ampicillin prestress in MC4100 and MG1655 strains. The survival ratio is shown in log scale. *Error bars* indicate the S.E. from three independent experiments. % survivals for all the strains are shown in supplemental Fig. S6.

The above studies involving overexpression of MazF suggest a possible role for the *mazEF* locus in the generation of persisters. Use of a conditionally expressible inactive, active-site mutant such as E24A MazF provides a powerful tool to conditionally regulate expression from the WT *mazEF* locus. However, the WT MazF levels generated using this system may not be physiologically relevant. We, therefore, studied MazF-mediated persister generation in the absence of an inactive, active-site mutant. For this purpose two different *E. coli* strains, BW25113 and MC4100, were initially used, and their survival was compared with the same strain having an identical genetic background but lacking the *mazF* locus. First, WT cells were exposed to a sublethal dose of either ampicillin (2.5 μg/ml, 1 h), ciprofloxacin (0.008 μg/ml, 1 h), or heat (48 °C, 20 min) followed by lethal doses of different antibiotics as used in previous experiments described above. Previously, MazF-mediated PCD was observed when MC4100 strain was exposed to rifampicin, spectinomycin, and various other antibiotics (38). Hence after prestress, MC4100 and MCΔmazF strains were exposed to cefotaxime, ciprofloxacin, and mitomycin C as well as to rifampicin (20 μg/ml) and spectinomycin (200 μg/ml), respectively. In a control experiment, cells were exposed to identical lethal doses of antibiotics without prestress. In both the strains ciprofloxacin prestress exposure resulted in a 2–10-fold higher survival against all antibiotics used relative to the corresponding strain lacking the *mazF* gene. In WT cells, even in the absence of a prestress, enhancement for persisters toward ciprofloxacin was observed (Fig. 7, *A* and *B*, supplemental Fig. S6, A and B). This shows that a basal level of MazF is able to induce cells at low frequency to a persistent state. No enhancement in persister frequency was observed in case of ampicillin and heat prestress. In contrast to the WT, pretreatment with a sublethal dose of antibiotics did not induce a higher frequency of persisters in both the Δ*mazF* strains. This shows that the *mazEF* locus is involved in the generation of persisters; however. the frequency of persisters induced by antibiotic prestress is lower relative to activation of WT MazF by overexpression of an inactive, active-site mutant. Both BW25113 and MC4100 strains have multiple deletions on the chromosome; hence, to study the role of MazF in persister formation, similar experiments were performed with an *E. coli* K-12 strain, MG1655, and its derivative MGΔmazF, which has a deletion of the *mazF* gene on the chromosome. Compared with the MGΔmazF strain, WT MG1655 strain showed a low but reproducible level of enhancement for persister formation against tobramycin (2-fold) and streptomycin (3-fold) when ciprofloxacin was given as prestress and against mitomycin C (10-fold) when cells were exposed to a sublethal dose of ampicillin (Fig. 7*C* and supplemental Fig. S6C). However, there was no enhancement in persister formation when cells were exposed to heat prestress or in the absence of prestress. Overall, some level of MazF-dependent protection against various antibiotics was seen in the case of all the *E. coli* strains used, although the effect is not as large as seen when using the overexpressed, inactive, active-site mutant E24A MaF. This difference could be because of a higher activation of the *mazEF* operon in the latter case.

##### Role of ClpP and Lon in MazF-mediated Persister Generation

**FIGURE 8:**
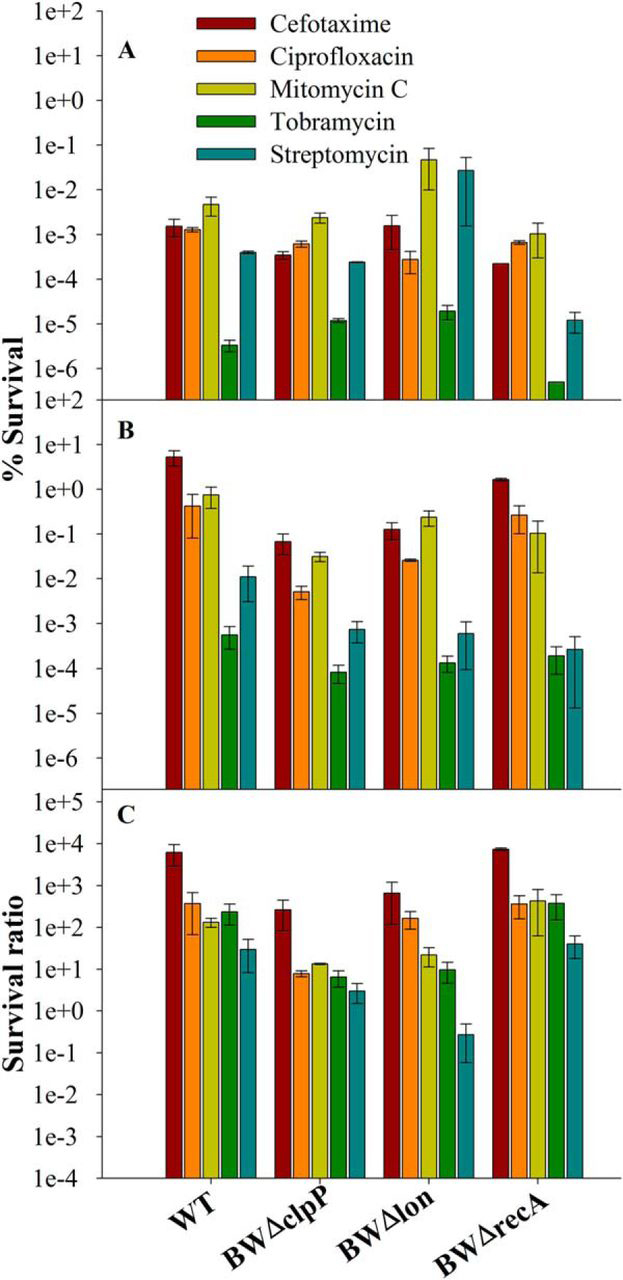
**Role of *clpP*, *lon*, and *recA* on MazF-mediated persister generation.** The strains WT (*E. coli* strain BW25113), BWΔclpP (BW25113 strain, lacks *clpP* gene), BWΔlon (BW25113 strain, lacks *lon* gene), and BWΔrecA (BW25113 strain, lacks *recA* gene) transformed with pBAD24E24AmazF plasmid were grown to an *A*_600_ of 0.2 and induced with 0.2% arabinose for 1 h. Subsequently cells were exposed to various antibiotics for 4 h, washed, and plated on 0.2% glucose containing LB-agar media. % survival was calculated as the ratio (cfu after antibiotic exposure/cfu before antibiotic exposure) ×100. Survival ratio is defined as (% survival of *E. coli* strain induced for E24A MazF/% survival of uninduced corresponding strain). *Panels A–C* show % survival of uninduced, induced cells, and survival ratio, respectively; both values are shown in log scale. *Error bars* indicate the S.E. from three independent experiments. Data show the decrease in survival in BWΔclpP and BWΔlon strains compared with WT but almost similar survival in WT and BWΔrecA, respectively. This indicates that MazF-mediated persister formation is dependent on ClpP and Lon proteases and independent of the presence of RecA.

In a bacterial cell, degradation of antitoxins by various proteases leads to activation of the corresponding toxins, which results in variety of phenotypes (3, 46, 47). In the TA pair, MazE-MazF, antitoxin MazE is a labile protein that is degraded by the ClpPA serine protease (10), although under conditions of nutritional starvation, a role for Lon was reported (3). Many antitoxins are also degraded by Lon protease including CcdA and RelB (48, 49). To study the effect of ClpP and Lon on MazF mediated-persister generation, an overexpressed inactive, active-site mutant, E24A MazF, was used. The *clpP* and *lon* genes were individually deleted from the BW25113 strain to yield the BWΔclpP and BWΔlon strains, respectively. The inactive, active-site mutant, E24A, was induced for 1 h in WT, BWΔclpP, and BWΔlon strains, and subsequently cells were exposed to lethal doses of various antibiotics as described above. Deletion of *clpP* and *lon* resulted in decreased persister formation with larger effects seen for ClpP. Approximately 35-, 40-, 10-, 40-, and 10-fold reduction in survival was observed for cefotaxime, ciprofloxacin, mitomycin C, tobramycin, and streptomycin, respectively, for Δ*clpP* relative to WT (Fig. 8), whereas about 8-, 2-, 5-, 20-, and 100-fold reduction in survival was observed for cefotaxime, ciprofloxacin, mitomycin C, tobramycin, and streptomycin, respectively, for Δ*lon* relative to WT (Fig. 8). In the absence of ClpP and Lon proteases, MazE is not degraded and, therefore, is able to inhibit WT MazF, thereby resulting in decreased persister formation. However, we do not see complete loss of persister formation, possibly because the overexpressed mutant, E24A MazF, titrates some of the MazE, resulting in liberation of some WT MazF. This also shows that proteolysis of MazE by ClpP and, to a lesser degree, Lon is an important factor in MazF-mediated persister generation. This is consistent with earlier studies that show the role for ClpP and Lon in MazE degradation during exponential growth and nutritional starvation, respectively (3, 10). The stability of MazE in WT BW25113 was compared with BWΔclpP and BWΔlon strains after heat induction at 48 °C to increase the cellular level of proteases. Degradation of MazE was observed in all three strains; however, degradation was higher in WT and *clp*-deleted strains. The reason for the lower stability for MazE in the *clp*-deleted strain could be that in our case, MazE has histidine and FLAG tags at the N and C termini, respectively, and both Lon and ClpP have different mechanisms for the recognition and degradation of peptide/proteins (50, 51). Hence, the addition of tags at the N or C terminus might have decreased/or abolished the affinity of ClpP for MazE for degradation (supplemental Fig. S7, A and B). Moreover, a decrease in persister frequency in BWΔclpP and BWΔlon strains relative to WT shows that proteolysis of MazE by ClpP and Lon is an important factor in MazF-mediated persister generation, and both proteases might be involved in MazE degradation or might have additional roles in downstream events after MazF activation.

##### MazF-mediated Persister Formation Is Independent of the Presence of RecA

It is known that exposure to bactericidal antibiotics such as ampicillin and ciprofloxacin results in RecA-mediated induction of SOS-response genes including DNA repair enzymes and Lon protease (52). RecA is a multifunctional protein that is activated either by a stalled replication fork or the presence of a single- and/or double-stranded DNA break and plays a role in DNA repair (53, 54). To study the effect of RecA on MazF-mediated persister generation, both BWΔrecA (BW25113 strain deleted for the *recA* gene) and corresponding WT strains were induced for E24A MazF. After this, cells were treated with various antibiotics. Survival was compared with corresponding uninduced strains. Although there is an expected decrease in overall survival in BWΔrecA relative to WT (9, 55), no significant difference in survival ratio was observed between the WT and BWΔrecA strains (Fig. 8). This shows that MazF-mediated persister generation is largely independent of the presence of RecA.

## DISCUSSION

TA loci are found both in bacterial and archaeal chromosomes and regulate growth under different conditions by modulating the global levels of translation and replication during nutritional stress and antibiotic exposure (2, 4, 7, 24, 56). There are different hypotheses available to explain the biological role of TA loci in growth, programmed cell death, persistence, and development (8, 17). The abundance of TA loci across various bacterial species indicates that they must have important roles in bacterial physiology and growth (7, 57). Multiple, apparently redundant TA modules are present in a bacterial cell. For example, the *M. tuberculosis* genome contains 88 putative TA systems (58). This redundancy hinders studying the function of a particular TA system. To understand the function of a TA locus, it is crucial to be able to specifically activate a particular TA locus in the presence of other TA loci. We have previously described an approach to study the function of a TA system by using an overexpressed, inactive, active-site mutant of the toxin component. This method was validated by studying the plasmid-based TA system, *ccdAB*, and elucidating a novel role for this in bacterial persistence (9). In the present study a similar approach was used to study the well characterized, chromosomal TA system, *mazEF.* Unlike the CcdAB system, for MazEF a homologous ChpAB system is also present in the cell. An inactive variant of MazF, E24A MazF was used. The residue Glu-24 which is required for activity, is located in a loop that is not involved in MazE binding and is disordered in the MazEF crystal structure (supplemental Fig. S1) (35). Hence, it is not surprising that mutations at this residue abolish MazF toxicity without affecting MazE binding. When this mutant was expressed in the wild type strain containing the chromosomal locus of *mazEF*, it resulted in growth arrest but not cell death. There was no effect when the same mutant was expressed in a Δ*mazF* strain. This demonstrated that activation of WT MazF by overexpression of the inactive, active-site mutant is responsible for the observed effects and is unaffected by the presence of the ChpAB homolog. Similar results were obtained in the background of BW25113, MC4100, and MG1655 strains. These observations support the growth modulation model (3, 16, 17) rather than the programmed cell death model of MazF function (10, 14).

We next probed the role of such reversible growth arrest in antibiotic persistence. Persisters are a dormant, slow growing fraction of an exponentially growing population of bacteria that are tolerant to lethal concentrations of antibiotics (21, 23, 24). The molecular mechanisms responsible for generation of persisters are poorly understood. Gene expression profiles of persisters have suggested roles for TA modules in persister generation (18, 20). In addition, ectopic expression of several different toxins such as HipA, RelE, MazF, MqsR, and TisB resulted in persistence (20, 24, 55, 59, 60). However, strains with *relBE* and *mazEF* TA modules did not show increased persister formation relative to strains deleted for the corresponding individual TA module (20), whereas deletion of genes for *tisB* from *E. coli* strain MG1655 and *mqsR* from BW25113 resulted in reduced persisters (55, 60). In the present study, when cells were exposed to various prestress, we observed a small but significant enhancement of persisters in all three WT *E. coli* strains, BW25113, MC4100, and MG1655 relative to the corresponding strain lacking the *mazF* gene (Fig. 7). Bacterial cells overproducing the toxins RelE or HipA undergo growth arrest and form 10–10,000-fold more persisters than in the absence of toxin overexpression (20). This observation suggests that the MazF-induced growth arrest in our system may be involved in persister formation, similar to observations found in other studies (18, 24). However, these earlier studies involve the ectopic overexpression of WT toxin in a cell from a heterologous promoter, which may not be physiologically relevant. To study the contribution of MazF in persistence, we use two different approaches. In the first approach WT MazF expression from its own promoter was activated by transient overexpression of the inactive, active-site mutant, E24A MazF. After this, substantial protection against cefotaxime, ciprofloxacin, mitomycin C, and tobramycin was seen, whereas no protection was observed against streptomycin (Fig. 4). Although this approach results in WT toxin being produced from its own promoter, it is likely that the resulting toxin levels may be higher than those resulting from activation of the TA system by naturally occurring stresses. Hence in the second approach, a variety of sublethal prestresses (heat or low levels of either ampicillin or ciprofloxacin) was used to activate the *mazEF* operon and survival after lethal antibiotic exposure was monitored. Ciprofloxacin prestress could consistently enhance survival ratio in both *E. coli* strains, BW25113 and MC4100. Heat prestress resulted in an enhanced survival ratio for cefotaxime and ciprofloxacin only in MC4100. This reflects differences in *mazEF* activation in these two different *E. coli* K12 strains. Compared with the BW25113 strain, MC4100 contains four deletions in the genome, ranging from 1 to 97 kb in size (61). Recently, a study has shown that arrested protein synthesis is one of the causes for persister generation, and the frequency of persisters can be enhanced by inhibiting protein synthesis by means of various chemicals including antibiotics such as rifampin and tetracycline (62). Similarly, inhibition of protein synthesis by MazF, when the *mazEF* system is activated at low levels either by means of an inactive, active-site mutant, or a sublethal dose of antibiotic, might be the cause for enhancement in persister frequency observed in the present work. The decreased protein synthesis can be observed by reduction in GFP expression (Figs. 2 and 3) upon MazF activation.

**FIGURE 9:**
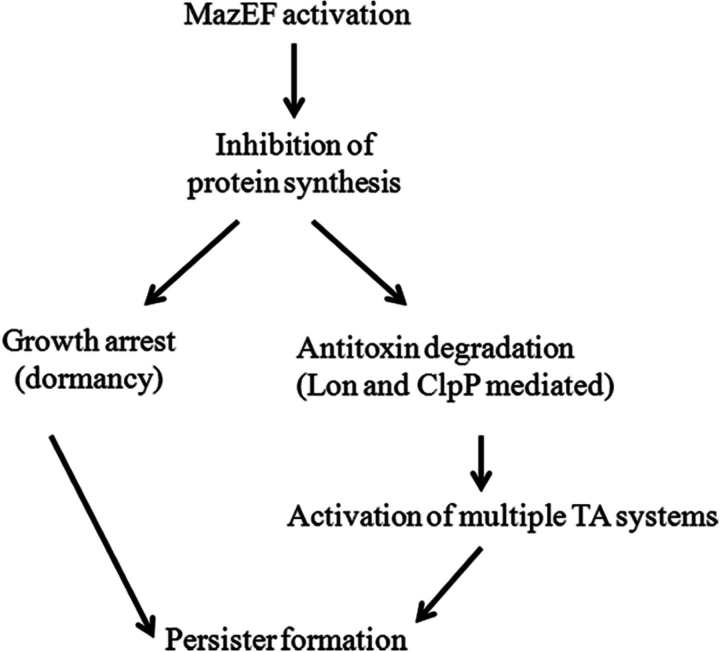
Schematic model for MazF-induced persister formation.

In bacterial cells, differential stability of antitoxin and toxin is found to affect the toxin-mediated persistence phenotype as shown for the HipBA and other chromosomal toxin-antitoxin systems (19, 63). The present study shows that the proteases ClpP and Lon, which are known to mediate degradation of the MazE antitoxin (3, 10), are also involved in MazF-mediated persister formation. However, unlike the situation for the CcdAB (9) and TisAB (55) TA systems, MazEF-mediated persister formation is surprisingly independent of RecA. A recent study (19) established the overall contribution of TA systems with endoribonuclease activity to persistence. However, multiple TA systems needed to be deleted before there was an observable decrease in persister frequency. In the present study, a direct contribution of the MazEF system to persister formation was detectable even in the background of other TA systems. A putative model for MazF-mediated persister generation is shown in Fig. 9. Cleavage of mRNA by MazF leads to global inhibition of protein synthesis, although recent work has also suggested some proteins can be preferentially translated after MazF expression (13, 64). Due to low stability of antitoxins relative to toxins, in the absence of fresh protein synthesis, antitoxin levels decrease because of cleavage by cellular proteases such as Lon and ClpP (3, 10, 19), thus leading to activation of multiple TA systems. In addition, decreased global protein synthesis leads to dormancy or growth arrest, which also contributes to multidrug tolerance (23, 62). A recent study has suggested that MazF preferentially cleaves in the antitoxin coding region of various TA mRNAs while leaving the toxin coding region intact, thus resulting in activation of multiple TA systems (65). All these factors collectively may contribute to MazF induced persister formation.

In conclusion, this work describes methodology involving use of an inactive variant of toxin for activation of a particular TA system, MazEF in the background of multiple TA systems. This work establishes the role of *E. coli* MazEF in growth arrest as opposed to programmed cell death and also clarifies the role of MazF arrested cells in bacterial persistence.

## Footnotes

3 The abbreviations used are:

TA: toxin-antitoxin
PCD: programmed cell death model.
