# Supplementary material for: MazF-induced Growth Inhibition and Persister Generation in Escherichia coli
Source: J Biol Chem. 2013 Dec 27;289(7):4191–205. doi: 10.1074/jbc.M113.510511 (PMC3924284; doi:10.1074/jbc.M113.510511)
Supplement: Supplementary file 1 [file mmc1.pdf]

SUPPLEMENTARY INFORMATION

MazF induced growth inhibition and persister generation in *Escherichia coli*

Arti Tripathi[a,d], Pooja C. Dewan[a,d] Shahbaz Ahmed[a] and Raghavan Varadarajan [a, b, c]

[a]Molecular Biophysics Unit, Indian Institute of Science, Bangalore 560 012, India

[b]Jawaharlal Nehru Center for Advanced Scientific Research, Jakkur P.O., Bangalore 560 004, India

***Running title**: MazF, bacteriostasis and persistence

[d]These authors contributed equally to this work
[c]To whom correspondence should be addressed: Molecular Biophysics Unit, Indian Institute of Science, Bangalore 560012, India. Tel.: +91-80-22932612. Fax: +91-80-23600535.
E-mail: *varadar@mbu.iisc.ernet.in*

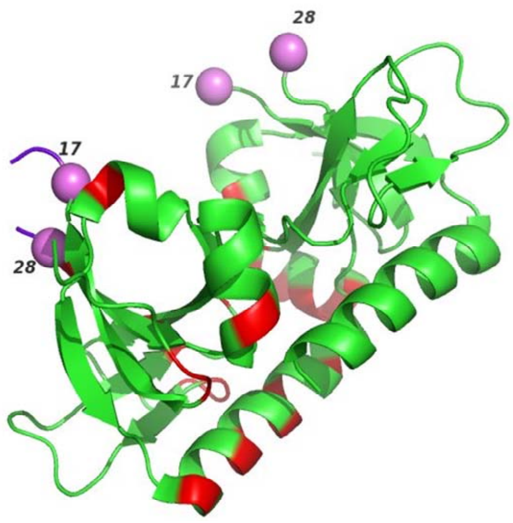

FIGURE S1. $MazF_2$ structure (green) showing residues interacting with MazE in red (PDB ID 1UB4, (1). Residues 17 and 28 from the A and B chains of $MazF_2$ are labeled and shown as violet spheres. The termini of the disordered loop (which contains residue E24) connecting 17 and 28 are shown in purple. This figure was created using PyMOL (http://www.pymol.org).

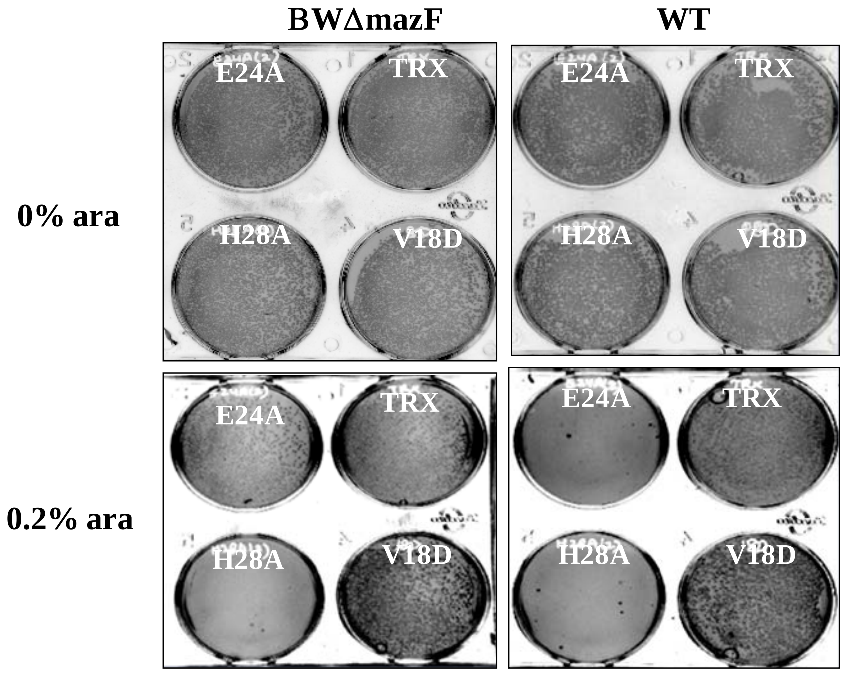

FIGURE S2. MazF inhibits cell growth only in WT cells when induced with 0.2 % arabinose. WT and BWΔmazF cells were transformed in parallel with plasmids expressing MazF mutants E24A and H28A. Following transformation and overnight growth to correct for differences in transformation efficiency, 50μl of a 10^{-4} dilution was plated on LB/amp plates containing 0% or 0.2% arabinose. Cells transformed with plasmid pBAD24 expressing TRX and V18D CcdB (2,3) were used as controls to show that over-expression of a non-toxic protein does not affect cell growth. Upon induction, the inactive mutant at the active-site of E24A MazF inhibits cell growth only in the WT strain while the active mutant H28A MazF inhibits cell growth in both strains.

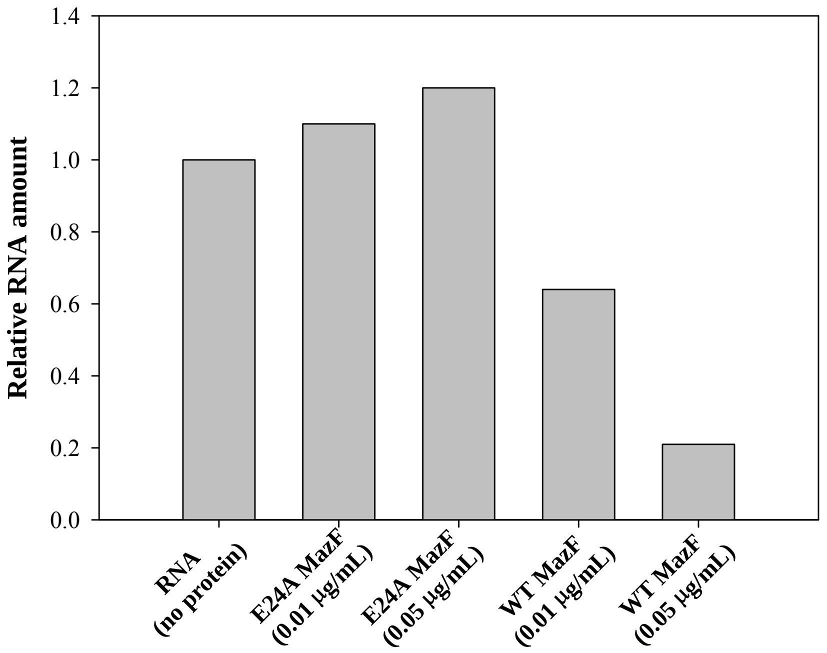

FIGURE S3. Endoribonuclease activity for WT MazF and E24A MazF. Total RNA from the *E.coli* strain MG1655 was purified and used as a substrate for MazF WT and E24A MazF respectively. 6 µg of RNA was incubated with 0.01 µg or 0.05 µg amount of MazF WT or E24A MazF at 37°C for 45 min in PBS buffer, pH7.4. E24A MazF shows no RNase activity in contrast to WT MazF.

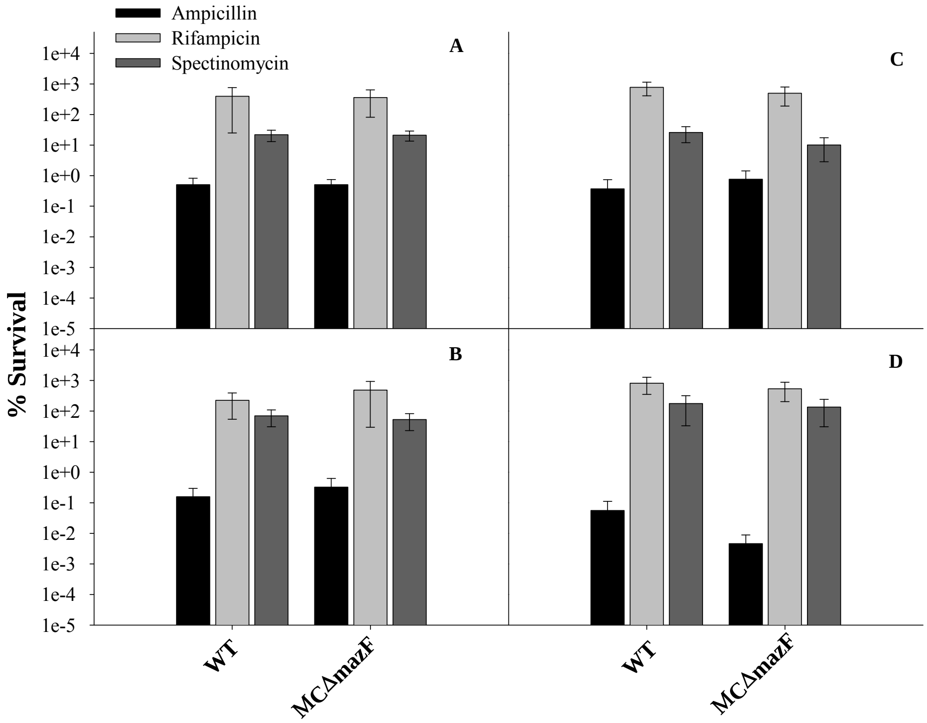

FIGURE S4. Deletion of MazF does not result in increased cell survival in presence of antibiotics. *E. coli* strains, WT (MC4100, contains *mazF* gene) and MCΔmazF (MC4100, lacks *mazF* gene) were grown in LB or M9 medium (4) with shaking (160 RPM) at 37°C to mid-logarithmic phase (OD_{600} of 0.4-0.6). An aliquot of the culture was withdrawn and tenfold serial dilutions were plated on LB-agar plates for counting initial number of cells for each strain. To each of the remaining cultures, ampicillin (100 μg/mL), rifampicin (15 μg/mL in M9 and 25 μg/mL in LB) or spectinomycin (200 μg/mL) was added and incubated for 10 min and 60 min respectively. Each sample was centrifuged at 4000 RPM for 5 min at room temperature and cells were washed twice with LB. Ten fold serial dilutions were prepared, plated on LB-agar plates and incubated at 37°C for 18 hours. % survival was defined as the ratio (CFU after antibiotic

exposure / CFU prior to antibiotic exposure) ×100. Panels A and B are % survival of cells after 10 min of antibiotic exposure in LB and M9 respectively and panels C and D are % survival of cells after 60 min of antibiotic exposure in LB and M9 media respectively. In presence of all the antibiotics and both at 10 and 60 min, strains WT and MCΔmazF show a similar pattern of growth in LB and M9 media respectively. The only exception is that in M9, medium WT strain shows 10-fold better growth in the presence of ampicillin, when incubated for 60 min.

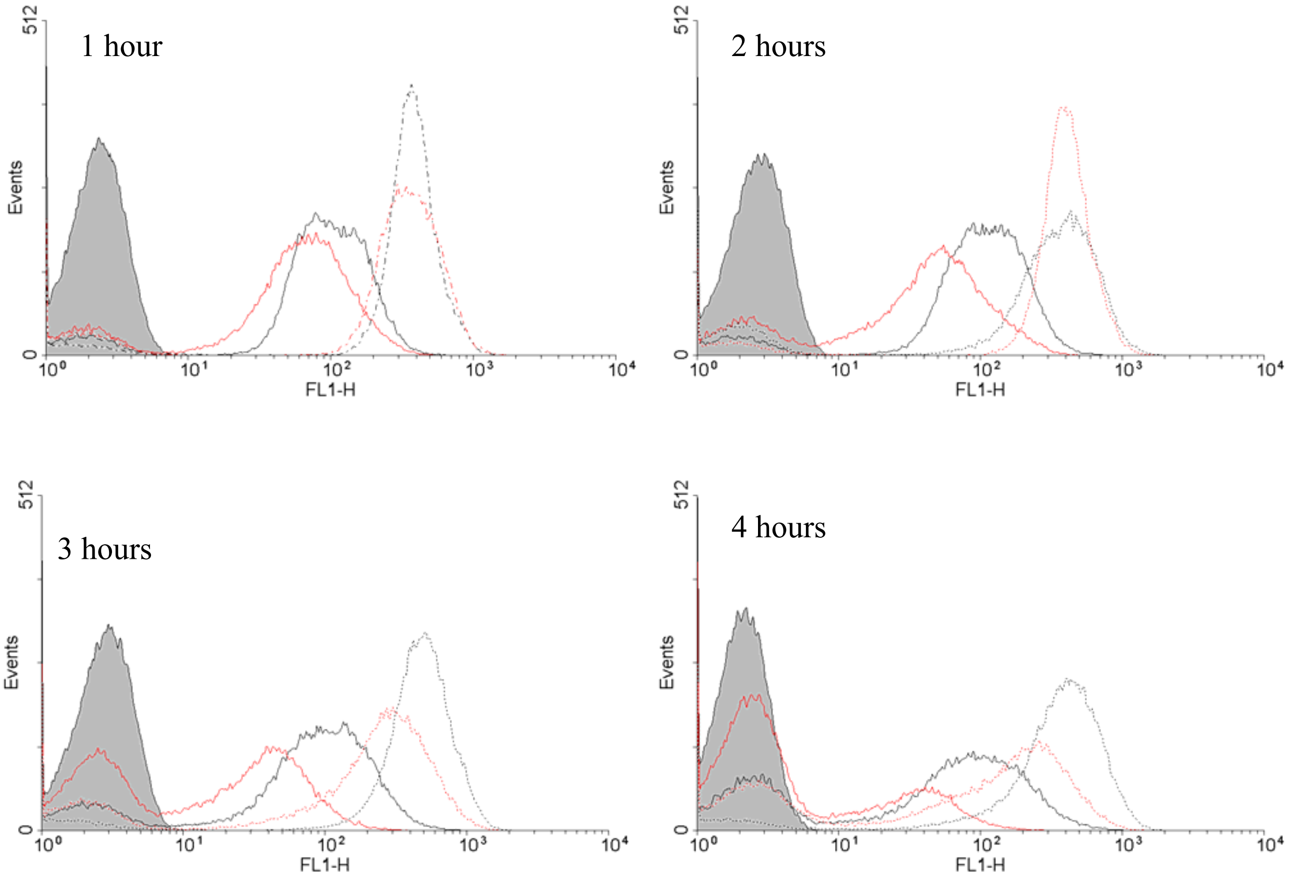

FIGURE S5. Effect of ciprofloxacin exposure on GFP expression in WT and *ΔmazF* strains, both expressing E24AMazF, monitored by FACS. Both WT and BWΔmazF strains, carrying the plasmid borne GFP and E24AMazF, were grown to an OD_{600} of 0.2, induced for an hour with 0.2% arabinose and subsequently treated with ciprofloxacin for 1-4 hours. GFP expression was monitored by flow cytometry at different time points. The black solid and dashed lines represent GFP expression in WT and BWΔmazF strains without ciprofloxacin treatment. The red solid and dashed lines represent GFP expression in WT and BWΔmazF strains with ciprofloxacin treatment. The filled grey curve represents GFP expression in the uninduced culture. The shaded

curve represents the uninduced control. There is a large time dependent increase in non-GFP expressing cells in WT cells relative to BWΔmazF cells following ciprofloxacin treatment. This shows that ciprofloxacin treatment causes a large fraction of cells to go into a dormant state in a MazF dependent manner.

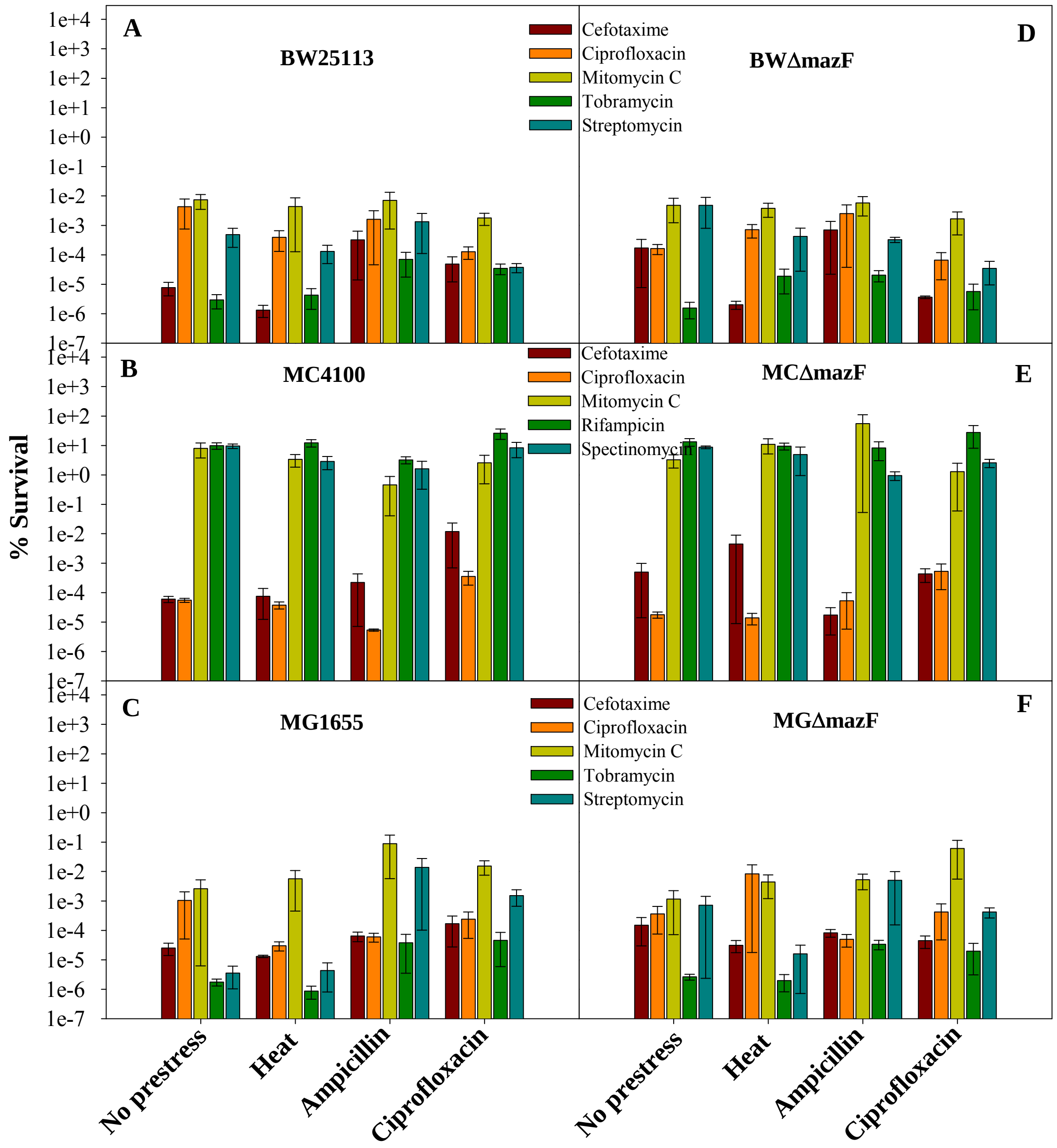

FIGURE S6. Role of MazF in persister generation when present in three different *E. coli* strains, BW25113, MC4100 and MG1655 respectively. Overnight culture of all *E. coli* strains, BW25113 (contains WT *mazF* gene), BWΔmazF (BW25113 strain, lacks *mazF* gene), MC4100 (contains WT *mazF* gene) and MCΔmazF (MC4100 strain, lacks *mazF* gene), MG1655 (contains WT *mazF* gene) and MGΔmazF (MG1655 strain, lacks *mazF* gene) were diluted 100 fold and

grown until an OD_{600} of 0.2-0.3. Subsequently, cells were exposed to two types of sublethal pre-stresses, either exposure of cells to heat (48°C for 20 min) or a sublethal dose of antibiotics (ampicillin (2.5 μg/mL for BW25113 and MC4100 and 5.0 μg/mL for MG1655) or ciprofloxacin (0.008 μg/ml)). This was followed by exposure of cells to various antibiotics at lethal doses (cefotaxime 100 μg/mL, ciprofloxacin 0.4 μg/mL, mitomycin C 10 μg/mL, tobramycin 25 μg/mL,streptomycin 25 μg/mL, rifampicin 20μg/mL, and spectinomycin 200 μg/mL) for 4 hours. Culture was washed twice with LB and plated on LB agar media for CFU/mL determination. % survival was defined as the ratio (CFU after antibiotic exposure / CFU prior to antibiotic exposure) ×100. Panels A, B and C show the % survival for WT BW25113, MC4100 and MG1655 strains respectively and panels D, E and F show the % survival for *ΔmazF* strains, BWΔmazF, MCΔmazF and MGΔmazF respectively. A sublethal dose of ciprofloxacin results in a small but significant increase in persister formation in all the *E. coli* strains, whereas heat pre-stresses enhance persisters only in MC4100 and ampicillin prestress in MC4100 and MG1655 strains. % Survival is shown in log scale. Error bars indicate the standard error from three independent experiments.

A

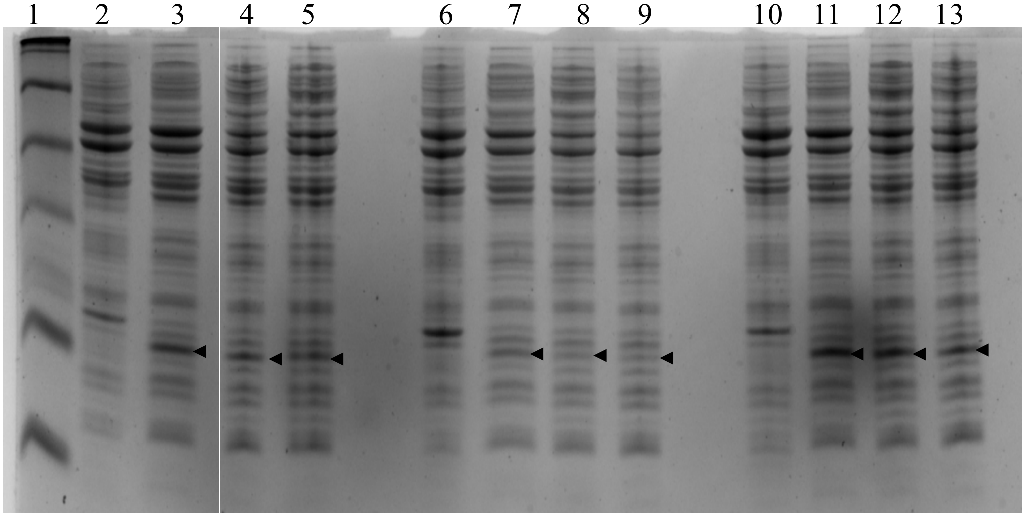

B

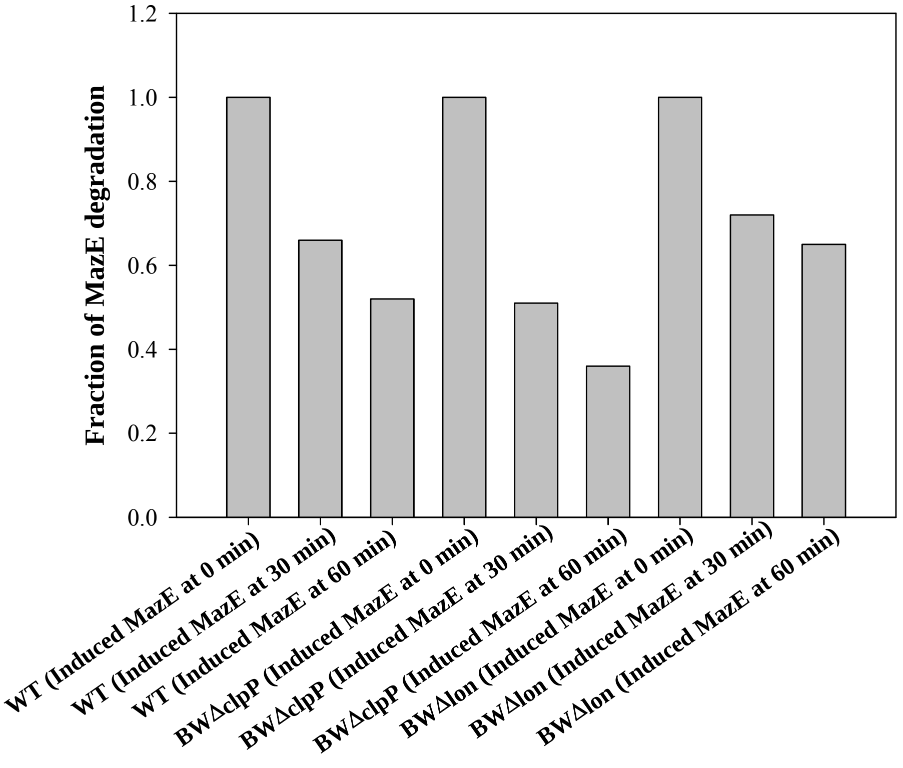

FIGURE S7. MazE stability in WT and protease deleted strains. WT (BW25113 strain), BWΔclpP (BW25113 lacking *clpP* gene) and BWΔlon (BW25113 lacking *lon* gene) strains were transformed with pBAD24mazE plasmid and grown in LB medium at 37°C, with shaking at 180

RPM to an OD_{600} of 0.5. Cells were induced with 0.001 % (w/v) arabinose for 2 hours. After 2 hours, inducer (arabinose) was removed and the repressor (glucose) was added at a final concentration of 2% (w/v) to repress the further expression of MazE from the P_{BAD} promoter. Heat shock was given at 48ºC for 20 min, followed by incubation at 37°C. Aliquots were taken at intervals of 30 min and the cellular level of MazE was monitored by SDS-PAGE and the extent of MazE degradation was quantitated using BioRad ChemiDoc^{TM} MP image software, Image Lab 4.1. Panel A shows the SDS-PAGE profile for MazE degradation in WT, BWΔclpP and BWΔlon strains. Symbol, ◄, indicates the induced protein band. Lane 1, protein marker; lane 2, uninduced WT; lane 3, WT induced for 2 hours; lane 4, WT induced for MazE for 2 hours , 20 min heat shock and 30 min incubation at 37°C; lane 5, WT induced for MazE for 2 hours , 20 min heat shock and 60 min incubation at 37°C; lane 6, uninduced BWΔclpP; lane 7, BWΔclpP induced for MazE for 2 hours; lane 8 BWΔclpP induced for MazE for 2 hours , 20 min heat shock and 30 min incubation at 37°C; lane 9, BWΔclpP induced for MazE for 2 hours , 20 min heat shock and 60 min incubation at 37°C; lane 10, uninduced BWΔlon; lane 11, BWΔlon induced for MazE for 2 hours; lane 12, BWΔlon induced for MazE for 2 hours , 20 min heat shock and 30 min incubation at 37°C; lane 13, BWΔlon induced for MazE for 2 hours , 20 min heat shock and 60 min incubation at 37°C. Panel B shows the quantitation of MazE degradation using Geldoc software. Induced band in each strain (lane 3, 7 and 11 for WT, BWΔclp and BWΔlon respectively) was taken as 1 and relative to induced band, fraction of MazE degradation was calculated after intervals of 30 and 60 min respectively. Stability of MazE is higher in BWΔlon strain relative to the WT and BWΔclpP strains, however, degradation of MazE is present in all the three strains. This shows that both ClpP and Lon proteases are responsible for the degradation of MazE.

REFERENCES

1. Kamada, K., Hanaoka, F., and Burley, S. K. (2003) Crystal structure of the MazE/MazF complex: molecular bases of antidote-toxin recognition. *Mol Cell* **11**, 875-884
2. Bajaj, K., Dewan, P. C., Chakrabarti, P., Goswami, D., Barua, B., Baliga, C., and Varadarajan, R. (2008) Structural correlates of the temperature sensitive phenotype derived from saturation mutagenesis studies of CcdB. *Biochemistry* **47**, 12964-12973
3. Tripathi, A., Dewan, P. C., Barua, B., and Varadarajan, R. (2012) Additional role for the ccd operon of F-plasmid as a transmissible persistence factor. *Proc Natl Acad Sci U S A*
4. Miller, J. H. (1972) *Experiments in molecular genetics*, Cold Spring Harbor Laboratory, Cold Spring Harbor, N. Y.
